# Joint modeling of longitudinal biomarker and survival outcomes with the presence of competing risk in the nested case–control studies with application to the TEDDY microbiome dataset

**DOI:** 10.1093/bioinformatics/btag038

**Published:** 2026-01-22

**Authors:** Yanan Zhao, Ting-Fang Lee, Boyan Zhou, Chan Wang, Ann Marie Schmidt, Mengling Liu, Huilin Li, Jiyuan Hu

**Affiliations:** Division of Biostatistics, Department of Population Health, NYU Grossman School of Medicine, New York, NY 10016, United States; Division of Biostatistics, Department of Population Health, NYU Grossman School of Medicine, New York, NY 10016, United States; Division of Biostatistics, Department of Population Health, NYU Grossman School of Medicine, New York, NY 10016, United States; Division of Biostatistics, Department of Population Health, NYU Grossman School of Medicine, New York, NY 10016, United States; Department of Medicine, NYU Langone Health, New York, NY 10016, United States; Division of Biostatistics, Department of Population Health, NYU Grossman School of Medicine, New York, NY 10016, United States; Division of Biostatistics, Department of Population Health, NYU Grossman School of Medicine, New York, NY 10016, United States; Division of Biostatistics, Department of Population Health, NYU Grossman School of Medicine, New York, NY 10016, United States

## Abstract

**Motivation:**

Large-scale prospective cohort studies collect longitudinal biospecimens alongside time-to-event outcomes to investigate biomarker dynamics in relation to disease risk. The nested case–control (NCC) design provides a cost-effective alternative to full cohort biomarker studies while preserving statistical efficiency. Despite advances in joint modeling for longitudinal and time-to-event outcomes, few approaches address the unique challenges posed by NCC sampling, non-normally distributed biomarkers, and competing survival outcomes.

**Results:**

Motivated by the TEDDY study, we propose “JM-NCC”, a joint modeling framework designed for NCC studies with competing events. It integrates a generalized linear mixed-effects model for potentially non-normally distributed biomarkers with a cause-specific hazard model for competing risks. Two estimation methods are developed. fJM-NCC leverages NCC sub-cohort longitudinal biomarker data and full cohort survival and clinical metadata, while wJM-NCC uses only NCC sub-cohort data. Both simulation studies and an application to TEDDY microbiome dataset demonstrate the robustness and efficiency of the proposed methods.

**Availability and implementation:**

Software is available at https://github.com/Zhaoyn-oss/JMNCC and archived on Zenodo at https://zenodo.org/records/18199759 (DOI: 10.5281/zenodo.18199759).

## 1 Introduction

The human microbiota is a dynamic community of microorganisms that inhabit various body niches, including the oral cavity, colon, and skin (Ursell *et al.* 2012). Alterations in microbiota composition can significantly impact health, potentially predisposing individuals to immunological and pathological conditions (Eckburg *et al.* 2005, Peterson *et al.* 2009, Blaser 2010). Advances in next-generation sequencing technologies, such as 16S rRNA and shotgun metagenomic sequencing, have facilitated in-depth exploration of the human microbiome’s role in diseases, including type 1 diabetes (T1D) (Needell and Zipris 2016, Paun *et al.* 2017, Vatanen *et al.* 2018, Dedrick *et al.* 2020), inflammatory bowel disease (Lloyd-Price *et al.* 2019, Fornelos *et al.* 2020, Zhang *et al.* 2022), and cancer (Rajagopala *et al.* 2017, Jiang *et al.* 2021, Jeong *et al.* 2024).

Large-scale cohort studies, such as the Integrative Human Microbiome Project (iHMP) (Integrative HMP 2019) and the Environmental Determinants of Diabetes in the Young (TEDDY) biomarker study, where gut microbiome is one of the key biomarker profiles (Hagopian *et al.* 2011, Lee *et al.* 2014, Stewart *et al.* 2018, Vatanen *et al.* 2018), offer unique opportunities to explore microbial biomarker–disease relationships in well-characterized human populations. However, large cohort size and high sequencing cost make the full cohort biomarker study both expensive and inefficient. To address this, TEDDY study adopted a nested case–control (NCC) design, in which only biospecimen from the NCC sub-cohort were processed to evaluate the corresponding biomarkers in relation to IA (islet autoimmunity)/T1D (type 1 diabetes) onset (Rundle *et al.* 2005, 2012, Lee 2014). NCC design offers a cost-efficient solution for large-scale biomarker studies, while also introducing unique analytical challenges for downstream statistical modeling.

While monthly to quarterly gut microbiome data are available in the TEDDY NCC sub-cohort, TEDDY study group has primarily analyzed these data using conditional logistic regression (CLR) (Stewart *et al.* 2018, Vatanen *et al.* 2018). This approach is specifically for matched case–control design studies and does not account for longitudinal measurements. Associations between microbial diversity or abundance at each sampling time points and dichotomous case–control status were examined, and limited microbial associations with IA or T1D were detected. This highlights an urgent need for methods capable of jointly modeling longitudinally sampled, non-normally distributed microbial biomarker profiles and their temporal trajectories alongside the time to onset of IA/T1D, while accounting for the NCC design. Furthermore, The TEDDY study group reported the heterogeneity of T1D by categorizing IA into distinct phenotypes based on the first appearing autoantibody (IAA, GADA, IA2A) (Krischer *et al.* 2015, 2017, Expert Committee on the Diagnosis and Classification of Diabetes Mellitus 2003), underscoring the need for methods that account for competing risks in survival analysis to model the heterogeneity.

Several analytical methods have been developed for NCC studies, including the standard conditional logistic regression model for matched case–control data (Gail *et al.* 1981) that the previous TEDDY microbiome association analyses have employed (Stewart *et al.* 2018, Vatanen *et al.* 2018), and refined methods such as the inverse selection probability weighting method (Samuelsen 1997), the local averaging method (Chen 2001), and likelihood-based methods (Scheike and Juul 2004, Zeng *et al.* 2006). However, these methods cannot accommodate time-varying covariates such as repeatedly measured microbiome data generated by TEDDY. Joint modeling frameworks, which integrate longitudinal covariates and time-to-event outcomes, provide a promising alternative (Hogan and Laird 1997, Tsiatis and Davidian 2004, Papageorgiou *et al.* 2019,  Goodrich *et al.* 2025, Afonso *et al.* 2023, Rustand *et al.* 2024). However, special considerations are required to extend such frameworks to NCC designs. Tseng and Liu (2009) first proposed a joint modeling approach for NCC studies, constructing a likelihood function based on all observed data to assess the relationships between longitudinal covariates and time-to-event outcomes. San (2013) extended this approach by focusing on NCC sub-cohort data only and employing an inverse probability weighting likelihood function for parameter estimation. Both methods are limited by their reliance on normality assumptions for longitudinal data and their inability to handle competing risks survival outcomes.

To our knowledge, no current method adequately addresses the following challenges: (i) characterizing longitudinally measured, non-normally distributed microbial biomarker data, (ii) accommodating the NCC design while leveraging clinical metadata and survival outcome data from the full cohort, (iii) modeling competing patterns in the appearance of autoantibodies.

**Figure 1:**
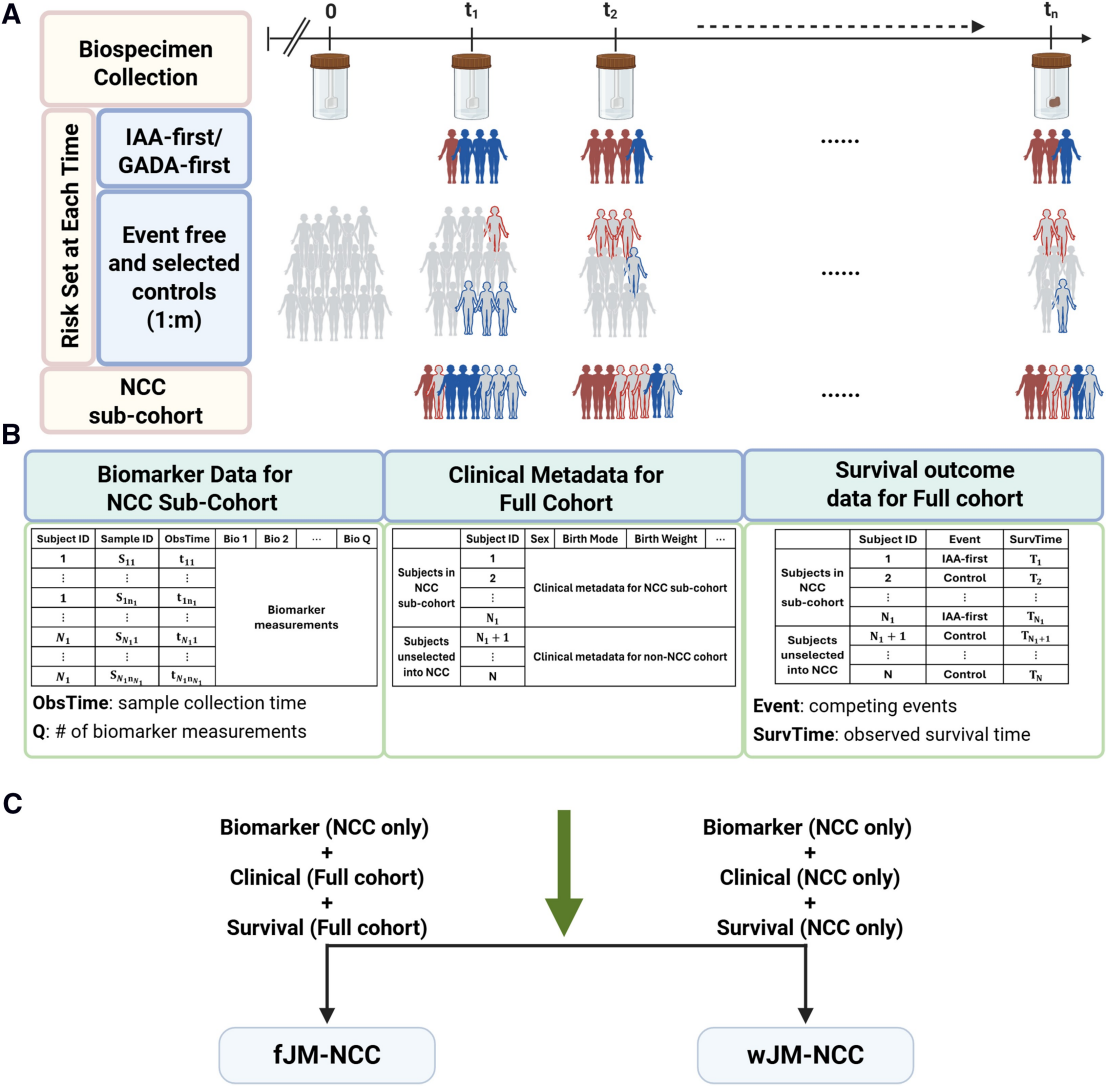
Overview of the nested case–control (NCC) study design and data used by fJM-NCC and wJM-NCC, using TEDDY biomarker study as an example. (A) Full cohort biospecimen collection and construction of the NCC sub-cohort based on 1:m ratio. (B) Analyzed data consisting of three main components: longitudinal biomarker data available only in NCC sub-cohort, clinical metadata, and survival outcomes for the full cohort. (C) Data requirements for fJM-NCC and wJM-NCC. The fJM-NCC method requires NCC biomarker data, full cohort clinical data and survival outcomes while the wJM-NCC method only requires data from the NCC sub-cohort.

Motivated by the unique TEDDY microbiome study, we propose a joint modeling method (JM-NCC) for analyzing longitudinal microbial biomarkers and competing events under NCC sampling. JM-NCC comprises two sub-models: a generalized linear mixed-effects model (longitudinal sub-model) to model biomarker trajectories over time, and a cause-specific hazard model (survival sub-model) to link these trajectories to competing events. Two inference approaches, i.e. fJM-NCC and wJM-NCC, are proposed depending on the availability of full cohort clinical metadata. Figure 1 illustrates the NCC study design with TEDDY biomarker study as an example, and the data utilized by fJM-NCC and wJM-NCC.

## 2 Materials and methods

### 2.1 Notation and model specification

#### 2.1.1 Full cohort

We consider a prospective cohort where the study outcomes are competing events, and longitudinal, high-dimensional microbial biomarkers are evaluated within a sub-cohort selected via NCC sampling. Let the cohort consist of N subjects. Following the notation of Tseng and Liu (2009), let $T_{ik}^{*}$ represent the time to competing event *k* (where k=1, 2,…, K), and $C_{i}$ denote the censoring time for subject i ( where i=1, 2,…, N). The observed survival time is defined by the observed survival time $T_{i}\equiv \min\left(C_{i},T_{i1}^{*},\;\ldots ,\;T_{iK}^{*}\right),$ with the censoring indicator $\delta_{i}\in \{0,\;1,\;\ldots ,\;K\}$, where $\delta_{i}=0$ represents censoring, and $\delta_{i}=k$ specifies that subject i experienced competing event k. Baseline clinical metadata (covariates) for each subject i are denoted by $X_{i}={\left(X_{i1},\;X_{i2},\;\ldots ,X_{ip}\right)}^{T}$ of length p. The full cohort data are thus denoted as $\left\{{\left(T\right)}_{i},\;\delta_{i},\;X_{i};i=1,\;2,\;\ldots ,\;N\right\}$.

#### 2.1.2 NCC sub-cohort

All subjects with $\delta_{i}\neq 0$ are further included in the NCC sub-cohort as cases. For each case i, m (≥1 denotes the control-to-case ratio) controls are randomly selected from the corresponding risk set, consisting of individuals who have not experienced any event by time $T_{i}$ and match case i on specific matching factors. Let the NCC sub-cohort consist of $N_{1}\;(N_{1}<N)$ subjects. The statistical efficiency of the NCC study depends on the number of controls selected per case (m), with increasing numbers of controls approaching the full cohort statistical power. Longitudinal high-dimensional ′-omics biomarker data are collected only for subjects in the NCC sub-cohort to retain study power while minimizing costs. These biomarkers are denoted as $Y_{i}={\left(Y_{i1},\ldots ,Y_{in_{i}}\right)}^{T},\;$measured at times $t_{i}={\left(t_{i1},\;t_{i2},\ldots ,\;t_{in_{i}}\right)}^{T}$, where $t_{in_{i}}\leq T_{i}$. Using an indicator variable $R_{i}=1$ for subject i included in the NCC sub-cohort ($R_{i}=0$ otherwise), the observed data for the NCC sub-cohort are denoted as $\left\{(T_{i},\;\delta_{i},\;X_{i},Y_{i},t_{i});\;R_{i}=1\right\}$.

We propose a joint model framework JM-NCC for longitudinal and competing event outcomes under NCC sampling. JM-NCC comprises two sub-models: a generalized linear mixed-effects model for capturing the temporal trajectory of longitudinal biomarker profiles (the longitudinal sub-model) and a cause-specific hazard model (Lau *et al.* 2009) for competing outcomes (the survival sub-model).

#### 2.1.3 Longitudinal sub-model

We first model the change of longitudinal biomarker measurements over time using the generalized linear mixed-effects model to account for non-normally distributed biomarker data. Noticeably, the proposed framework considers each biomarker separately, and therefore we redefine $Y_{i}={\left(Y_{i1},\ldots ,Y_{in_{i}}\right)}^{T}$ to denote the longitudinal measurement of a specific biomarker at time $t_{i}={\left(t_{i1},\ldots ,t_{in_{i}}\right)}^{T}$ for simplicity. For a given biomarker, we assume the abundance of the jth sample $Y_{ij}$ follows an exponential family distribution of $f(\mu_{ij},\;a_{ij}(\tau )\upsilon (\mu_{ij}))\;$conditional on random effects $b_{i}$ detailed below, where $\mu_{ij}=E[Y_{ij}|b_{i}]$ is the conditional mean of $Y_{ij}$ given $b_{i}$, τ is an unknown dispersion parameter, $a_{ij}(\cdot )$ and υ(·) are known functions (Dean and Nielsen 2007, Jiang and Nguyen 2007). Then we have


(1) g(E[Yij|bi])=γTXi(1)+biT Zi,

where g(⋅) is the link function relating the linear predictor $\gamma^{T}X_{i}^{\left(1\right)}+b_{i}^{T}{\;Z}_{i}$ to the expectation $E[Y_{ij}|b_{i}]$, $X_{i}^{\left(1\right)}$ and γ correspond to the fixed effects design matrix that including times $t_{i}$ and a subset of baseline covariates, and coefficients, while $Z_{i}$ and $b_{i}$ denote the random effects design matrix and subject-specific random effects. Random effect $b_{i}\;$are assumed to follow a multivariate normal distribution $N(0,\;\Sigma_{\theta })$. Sub-model (1) simplifies to a linear mixed-effects model when $Y_{ij}$ is assumed to follow a normal distribution $N(\mu_{ij},\;\sigma^{2})$, with parameters $a_{ij}(\tau )=\sigma^{2}$ and $\upsilon (\mu_{ij})=1$. For read count data, we can model $Y_{ij}$ using a Poisson generalized linear mixed-effects model with a log link and subject-specific random effects: $Y_{ij}\;|b_{i}∼\;\mathrm{Poisson}(\mu_{ij})$, with ${\log\left(\mu \right)}_{ij}=\gamma^{T}X_{i}^{\left(1\right)}+b_{i}^{T}{\;Z}_{i}.$

#### 2.1.4 Survival sub-model

We use a cause-specific hazard model to estimate associations between longitudinal measurements and competing events:


(2) $$\begin{array}{c} \lambda_{k}(t|b_{i},Y_{i},\;t_{i},\;X_{i})=\lambda_{0k}(t)\exp\left(\beta_{k}\cdot g^{-1}(\gamma^{T}X_{i}^{\left(1\right)}+b_{i}^{T}{\;Z}_{i})+\alpha_{k}^{T}\cdot X_{i}^{\left(2\right)}\right),\\ k=1,\ldots ,K; \end{array}$$

where $\lambda_{0k}(t)$ is the baseline cause-specific hazard function for competing event k (k=1, 2,…, K), $g^{-1}(\cdot )$ is the inverse of link function g(⋅), and $X_{i}^{\left(2\right)}$ represents the subset of baseline covariates associated with the competing events. The regression coefficients $\beta_{k}\;(k=1,\ldots ,K)$ are parameters of interest quantifying the association between the temporal trajectory of the assessed biomarker and competing event k, while $\alpha_{k}$ represents the association between the covariates $X_{i}^{\left(2\right)}$ and competing event k. The longitudinal covariates in $X_{i}^{\left(1\right)}$ may overlap with survival covariates $X_{i}^{\left(2\right)}$, the interpretation of $\alpha_{k}$ can be considered as the effect of $X_{i}^{\left(2\right)}$ on competing event k, conditional on the true value of $Y_{i},$ which is $g^{-1}(\gamma^{T}X_{i}^{\left(1\right)}+b_{i}^{T}{\;Z}_{i})$. For K=1, this model reduces to a Cox PH model for a general time-to-event outcome.

Let $\Phi =({\gamma^{T},\;\tau ,\mathrm{vech}(\Sigma_{\theta }),\lambda_{0k}(t),\;\beta }_{k},\alpha_{k}^{T})$ be the set of all parameters to be estimated, where vech(⋅) denotes the half-vectorization of a matrix. The baseline hazard function $\lambda_{0k}(t)\;$is modeled using a piecewise-constant approach: $\lambda_{0k}(t)=\sum_{q=1}^{Q}\xi_{kq}I(\nu_{q-1}<t{\leq \nu }_{q})$, where ${0=\nu }_{0}<\nu_{1}\cdots {<\nu }_{Q}$ denotes a split of the time scale and $\xi_{kq}$ represents the hazard value for competing k within $\left(\nu_{q-1},\;\nu_{q}\right)$. We split the parameter set Φ into two components, the parametric component $\phi =({\gamma^{T},\tau ,\mathrm{vech}(\Sigma_{\theta }),\;\beta }_{k},\alpha_{k}^{T})$ and the collection of non-parametric baseline hazard $\Lambda_{k}=(\xi_{k1},\ldots ,\;\xi_{kQ})$, where our primary interest lies in making inferences about ϕ.

We present two MLE approaches for estimating ϕ, referred to as fJM-NCC and wJM-NCC respectively. The fJM-NCC method constructs the likelihood function by integrating longitudinal measurements from the NCC sub-cohort with survival data and clinical metadata from the full cohort. However, in many biomarker studies, certain clinical metadata, such as diet and blood concentrations of nutrient biomarkers in the TEDDY study, are not accessed for all subjects in the full cohort. To address this, the wJM-NCC method builds an inverse probability weighting likelihood function using only the data from NCC sub-cohort for parameter estimation.

### 2.2 fJM-NCC: likelihood inference with full maximum likelihood approach

The fJM-NCC method incorporates all observed data and survival outcome to estimate ϕ, treating the unobserved longitudinal measurements as missing at random. The full log-likelihood function is given by


$$l_{\mathrm{full}}(\phi )=\sum_{i=1}^{N}\log\int f(T_{i},\delta_{i},Y_{i}|b_{i},X_{i})f(b_{i})db_{i},$$

where $f(T_{i},\;\delta_{i},\;Y_{i}|b_{i},X_{i})$ is the conditional probability density function (pdf) of $\left(T_{i},\;\delta_{i},\;Y_{i}\right)$ given $b_{i}\;$and $X_{i}$, $f(b_{i})\;$is the pdf of $b_{i}$. Assuming the longitudinal and time-to-event data-generating processes are conditionally independent given$\;b_{i}$, the joint density simplifies to $f(T_{i},\delta_{i},Y_{i}|b_{i},X_{i})=f(T_{i},\;\delta_{i}|b_{i},X_{i})f(Y_{i}|b_{i},X_{i})$. For subjects with unobserved $Y_{i}$, $\left\{i;R_{i}=0\right\}$, $f(Y_{i}|b_{i},X_{i})=1$. Therefore, the full log-likelihood function can be rewritten as


(3) $$l_{\mathrm{full}}(\phi )=\sum_{i;R_{i}=0}\log\int f(T_{i},\delta_{i}|b_{i},X_{i})f(b_{i})db_{i}+\sum_{i;R_{i}=1}\log\int f(T_{i},\delta_{i}|b_{i},X_{i})f(Y_{i}|b_{i},X_{i})f(b_{i})db_{i}.$$

Gaussian quadrature is employed to approximate the integral in Equation (3), where detailed derivation of the numerical approximation is shown in Supplementary Materials, Section S1, available as supplementary data at *Bioinformatics* online. The MLEs of ϕ are obtained by maximizing the full log-likelihood function (3). The standard errors (SEs) of parameters are derived using the Fisher’s information matrix, which is consistently estimated by the empirical Fisher’s information matrix. The 95% confidence intervals (CIs) of the parameters can be calculated as ${\hat{\phi }}_{\mathrm{full}}\pm 1.96\;\mathrm{SE}({\hat{\phi }}_{\mathrm{full}})$. The Wald test statistic (Gregory and Veall 1985) is used to test this null hypothesis for each competing event k: $H_{0}:\beta_{k}=0$.

### 2.3 wJM-NCC: likelihood inference with the inverse probability weighting approach

The wJM-NCC method constructs an inverse probability weighting likelihood function using only data from the NCC sub-cohort, accounting for the potential selection bias in the NCC sampling. Such bias arises because subjects who remain in the full cohort for longer duration are more likely to be included in the NCC sub-cohort. The weighted log-likelihood function is given by:


(4) $$l_{\mathrm{wt}}(\phi )=\sum_{i;R_{i}=1}w_{i}\log\left(\int f(T_{i},\;\delta_{i}|b_{i},X_{i})f(Y_{i}|b_{i},X_{i})f(b_{i})db_{i}\right),$$

where weight


$$w_{i}={\left(1-\prod_{l\in S_{i}}\left(1-\frac{m_{l}}{n_{l}}\right)\right)}^{-1}$$

presents the inverse probability of inclusion as a control. $S_{i}$ is the set of cases for which subject i was eligible to be selected as a control, $m_{l}$ is the number of controls selected for case l, and $n_{l}$ is the number of candidates in the risk set for case l. Notably, $w_{i}=1$ if subject i is a case. As in the full log-likelihood function, the integral component in Equation (4) is approximated using Gaussian quadrature to obtain the MLE of parameter ϕ (see Supplementary Materials, Section S1, available as supplementary data at *Bioinformatics* online).

#### 2.3.1 Standard error estimation

Direct application of the Fisher’s information matrix to the weighted log-likelihood function tends to underestimate the standard errors (San 2013). To address this, we employed the sandwich method to derive a robust covariance estimator for the weighted log-likelihood function (4). The sandwich covariance estimator is given by


$$\mathrm{Cov}(\phi )=I^{-1}(\phi )\Sigma (\Phi )I^{-1}(\phi ),$$

where


$$I(\phi )=-E[\frac{\partial^{2}}{\partial \phi \partial \phi^{T}}l_{\mathrm{wt}}(\phi )],\Sigma (\phi )=\mathrm{cov}[\frac{\partial }{\partial \phi }l_{\mathrm{wt}}(\phi )].$$

I(ϕ) and Σ(ϕ) can be consistently estimated by the empirical Fisher’s information matrix and the empirical covariance matrix, respectively (see Supplementary Materials, Section S2, available as supplementary data at *Bioinformatics* online). The 95% CIs for the parameters and hypothesis tests are conducted in the same manner as for fJM-NCC.

## 3 Results

### 3.1 Simulation studies

#### 3.1.1 Simulation setup

We conducted three simulation studies to comprehensively evaluate the inference performance of the two proposed methods compared with competing methods.

##### 3.1.1.1 Study 1: simple longitudinal structure and constant baseline hazards

A single longitudinal biomarker was simulated from a linear mixed-effects model with a fixed slope and a subject-specific random intercept. Two competing events were generated using the cause-specific hazard model with constant baseline hazard functions for each competing event. We considered two scenarios to assess inference performance for the association parameters $\beta_{1}$ and $\beta_{2}$: (1) Scenario 1 (global null, ${\beta_{1}=\beta }_{2}=0$): no association between longitudinal trajectory and either competing event; (2) Scenario 2 (alternative): fixing $\beta_{2}=0.1$ and varying $\beta_{1}$ from 0 to 0.3 in increments of 0.1, allowing us to assess power and estimation accuracy under increasing effect sizes.

##### 3.1.1.2 Study 2: complex longitudinal structure and piecewise-constant baseline hazards

In Study 2, we considered a more complex longitudinal process that included both random intercept and random slope terms in the longitudinal model. The baseline hazard functions for competing events were specified as piecewise-constant to allow time-varying event risks.

##### 3.1.1.3 Study 3: high-dimensional longitudinal microbiome biomarker data

In Study 3, we used SparseDOSSA2 (Ma *et al.* 2021) to generate 100 longitudinal microbial taxa abundances, where 10% of the taxa were randomly selected as true signals with effect sizes $\beta_{1}=0.5$ and $\beta_{2}=-0.5$ for the two competing events. The absolute abundances generated were log-transformed prior to model fitting for all competition methods. In fJM-NCC and wJM-NCC, linear mixed effects model was adopted as the longitudinal sub-model accordingly.

To approximate the event rate of islet autoimmunity (IA) observed in the TEDDY study (Tsiatis and Davidian 2004), survival times were simulated for a full cohort of N=8000 subjects for three studies. A dichotomous baseline covariate $X_{i}^{\left(2\right)}∼\mathrm{Bernoulli}(0.5)$ was included in the survival sub-model. The first 400 subjects experiencing either event 1 or event 2 were selected to form the cases in the NCC sub-cohort. In order to form 400 cases in the NCC sub-cohort, a censoring framework, consisting of two sequential censoring steps, was applied: Step (1) Random censoring: censoring times were drawn from a uniform distribution to mimic general right-censoring; Step (2) Administrative censoring: the censoring time was set to the maximum event time among the first 400 observed cases. For each case, we randomly selected m = 1, 3, or 5 controls from the corresponding risk set, matched by gender $X_{i}^{\left(2\right)}$, to construct the NCC sub-cohort. Detailed data-generating mechanisms, parameter specifications, model settings, and competing methods [Oracle, wJM-NCC(Fisher), JM, and CLR] are provided in Supplementary Materials, Sections S3–S4, available as supplementary data at *Bioinformatics* online. Table 1 summarizes all methods considered in this article, outlining their data requirements, applicable study designs, and other relevant characteristics.

**Table 1:** Summary of data requirements and applicability of methods to NCC design, longitudinal biomarkers, and competing events.

Method	Full cohort data required^a^	Suitable for NCC design	Handles longitudinal biomarkers	Handles competing events	Brief description
Oracle	Yes	No	Yes	Yes	Benchmark method in simulation studies
fJM-NCC	Partial^b^	Yes	Yes	Yes	Outperforms other NCC methods in parameter estimation and hypothesis test efficiency
wJM-NCC	No	Yes	Yes	Yes	Standard error (SE) is estimated using the proposed Sandwich estimator
wJM-NCC(Fisher)	No	Yes	Yes	Yes	Variation of wJM-NCC; SE is estimated using Fisher’s information matrix
JM	Yes	No	Yes	Yes	Classical joint modeling approach for longitudinal and competing events outcomes; assumes normality of biomarkers.
CLR	No	Yes	No	No	Standard method for matched case–control study; uses mean biomarker value as covariates as it cannot handle time-varying covariates

a Full cohort data refers to the availability of clinical metadata, longitudinal biomarker measurements, and survival outcomes of all individuals in the full cohort.

b The fJM-NCC approach requires clinical metadata and survival data from the full cohort but only longitudinal biomarker data from the NCC sub-cohort.

#### 3.1.2 Simulation results

##### 3.1.2.1 Results of study 1

Table 2 summarizes the performance of point estimates and 95% confidence interval (CI) estimates for $\beta_{1}$ and $\beta_{2}$ under the global null hypothesis ($\beta_{1}=\beta_{2}=0$, Scenario 1). Across all methods, the point estimates for $\beta_{1}$ and $\beta_{2}$ are nearly unbiased. The SE estimates for both parameters closely match the ESEs across all methods except wJM-NCC(Fisher). The SEs from wJM-NCC(Fisher) are underestimated particularly when the number of controls per case is small (e.g. m=1). Accordingly, wJM-NCC(Fisher) produces shorter CIs due to SE underestimation, leading to lower empirical coverage rates (ECP) when m=1. In comparison, the newly proposed sandwich estimator in wJM-NCC provides well-calibrated SE estimates, which yield satisfactory coverage performance.

**Table 2:** Performance of all methods for point and 95% confidence interval estimation of $\beta_{1}$ and $\beta_{2}$ under Scenario 1 ($\beta_{1}=\beta_{2}=0$) in Study 1.

		$\beta_{1}$						$\beta_{2}$					
*m* ^a^	Method	Bias	SE^b^	ESE^c^	MSE^d^	CI-L^e^	ECP^f^	Bias	SE	ESE	MSE	CI-L	ECP
1	Oracle	−0.002	0.073	0.071	0.005	0.285	0.958	−0.001	0.044	0.046	0.002	0.172	0.929
	fJM-NCC	−0.003	0.074	0.073	0.005	0.292	0.959	−0.001	0.046	0.049	0.002	0.180	0.935
	wJM-NCC	−0.003	0.082	0.081	0.007	0.320	0.950	−0.002	0.057	0.060	0.004	0.225	0.939
	wJM-NCC(Fisher)	−0.003	0.073	0.081	0.007	0.285	0.920	−0.002	0.044	0.060	0.004	0.172	0.857
	JM	−0.003	0.072	0.072	0.005	0.284	0.958	−0.002	0.044	0.046	0.002	0.171	0.937
	CLR	−0.003	0.097	0.095	0.009	0.380	0.954	−0.004	0.058	0.059	0.004	0.226	0.942
3	Oracle	−0.001	0.072	0.072	0.005	0.284	0.954	0.001	0.044	0.043	0.002	0.172	0.957
	fJM-NCC	0.000	0.073	0.073	0.005	0.288	0.955	0.001	0.045	0.044	0.002	0.177	0.958
	wJM-NCC	0.000	0.075	0.075	0.006	0.293	0.961	0.001	0.048	0.047	0.002	0.190	0.960
	wJM-NCC(Fisher)	0.000	0.072	0.075	0.006	0.284	0.946	0.001	0.044	0.047	0.002	0.172	0.938
	JM	−0.001	0.072	0.071	0.005	0.282	0.955	0.001	0.044	0.043	0.002	0.171	0.960
	CLR	0.000	0.077	0.080	0.006	0.303	0.948	−0.001	0.047	0.046	0.002	0.183	0.957
5	Oracle	0.003	0.072	0.072	0.005	0.284	0.961	0.000	0.044	0.044	0.002	0.172	0.950
	fJM-NCC	0.002	0.073	0.074	0.005	0.287	0.957	0.000	0.045	0.045	0.002	0.176	0.960
	wJM-NCC	0.002	0.074	0.074	0.006	0.289	0.959	0.000	0.046	0.046	0.002	0.182	0.957
	wJM-NCC(Fisher)	0.002	0.072	0.074	0.006	0.284	0.951	0.000	0.044	0.046	0.002	0.172	0.942
	JM	0.002	0.072	0.072	0.005	0.282	0.956	−0.001	0.044	0.044	0.002	0.171	0.955
	CLR	0.000	0.073	0.073	0.005	0.287	0.953	−0.001	0.044	0.044	0.002	0.174	0.946

a Control-to-case ratio, i.e. the number of controls per case in the NCC sub-cohort.

b Estimated standard error.

c Empirical standard error.

d Mean squared error.

e Average length of the 95% confidence intervals.

f Empirical coverage probability of the 95% confidence interval.

Table 3 and Tables S1–S3, available as supplementary data at *Bioinformatics* online, present the results for point and 95% CI estimates for $\beta_{1}$ and $\beta_{2}$, with $\beta_{2}$ fixed at 0.1 and $\beta_{1}\;$varying from 0 (Table S1, available as supplementary data at *Bioinformatics* online), 0.1 (Table S2, available as supplementary data at *Bioinformatics* online), 0.2 (Table S3, available as supplementary data at *Bioinformatics* online), to 0.3 (Table 3) under Scenario 2. The point estimates for $\beta_{1}$ and $\beta_{2}$ from Oracle, fJM-NCC, and wJM-NCC remain unbiased with minimal bias, while JM shows considerable bias across all parameter settings. This bias arises because JM incorrectly treats the NCC sub-cohort as the full cohort, despite the event rate in the NCC sub-cohort being distorted to $\frac{1}{m+1}$. The bias decreases as the number of controls per case (m) increases. The CLR method provides unbiased point estimates for $\beta_{1}$, but exhibits substantial bias for $\beta_{2}$ when $\beta_{1}=0$ and $\beta_{2}=0.1$ (Table S1, available as supplementary data at *Bioinformatics* online), where the biomarker is only associated with one event. The bias in CLR’s point estimate for $\beta_{1}$ becomes more pronounced when biomarker value is associated with competing events (both $\beta_{1}$ and $\beta_{2}$ are non-zero; Table 3 and Tables S2 and S3, available as supplementary data at *Bioinformatics* online). SE estimates for $\beta_{1}$ and $\beta_{2}$ are consistent across Oracle, fJM-NCC, wJM-NCC, closely aligning with the ESEs. However, wJM-NCC(Fisher) continues to underestimate SEs. The CLR method produces the longest CIs in most settings, reflecting greater uncertainty in the parameter estimates compared to the other methods. Estimation results of nuisance parameters in the model are detailed in Supplementary Materials Section S5, available as supplementary data at *Bioinformatics* online.

**Table 3:** Performance of all methods for point and 95% confidence interval estimation of $\beta_{1}$ and $\beta_{2}$ under Scenario 2 ($\beta_{1}=0.3$ and $\beta_{2}=0.1$) in Study 1.

		$\beta_{1}$						$\beta_{2}$					
*m* ^a^	Method	Bias	SE^b^	ESE^c^	MSE^d^	CI-L^e^	ECP^f^	Bias	SE	ESE	MSE	CI-L	ECP
1	Oracle	−0.001	0.070	0.074	0.005	0.275	0.938	0.001	0.044	0.045	0.002	0.174	0.948
	fJM-NCC	0.003	0.074	0.078	0.006	0.289	0.943	0.002	0.047	0.048	0.002	0.184	0.951
	wJM-NCC	0.000	0.083	0.086	0.007	0.326	0.946	0.000	0.058	0.059	0.003	0.228	0.948
	wJM-NCC(Fisher)	0.000	0.070	0.086	0.007	0.276	0.904	0.000	0.045	0.059	0.003	0.175	0.865
	JM	−0.054	0.070	0.071	0.008	0.273	0.872	−0.048	0.044	0.045	0.004	0.172	0.797
	CLR	−0.022	0.101	0.103	0.011	0.396	0.936	−0.005	0.059	0.061	0.004	0.232	0.949
3	Oracle	0.004	0.070	0.070	0.005	0.274	0.957	0.003	0.045	0.044	0.002	0.175	0.955
	fJM-NCC	0.007	0.072	0.073	0.005	0.282	0.961	0.004	0.046	0.045	0.002	0.180	0.947
	wJM-NCC	0.005	0.074	0.074	0.006	0.290	0.955	0.002	0.049	0.048	0.002	0.193	0.955
	wJM-NCC(Fisher)	0.005	0.070	0.074	0.006	0.274	0.948	0.002	0.045	0.048	0.002	0.175	0.925
	JM	−0.023	0.069	0.069	0.005	0.272	0.945	−0.020	0.044	0.043	0.002	0.173	0.929
	CLR	−0.021	0.078	0.079	0.007	0.306	0.938	−0.005	0.048	0.047	0.002	0.187	0.949
5	Oracle	0.001	0.070	0.071	0.005	0.274	0.954	0.002	0.045	0.046	0.002	0.175	0.941
	fJM-NCC	0.003	0.071	0.072	0.005	0.280	0.950	0.002	0.046	0.048	0.002	0.179	0.939
	wJM-NCC	0.002	0.072	0.073	0.005	0.284	0.947	0.002	0.047	0.049	0.002	0.185	0.940
	wJM-NCC(Fisher)	0.002	0.070	0.073	0.005	0.274	0.947	0.002	0.045	0.049	0.002	0.175	0.922
	JM	−0.018	0.069	0.070	0.005	0.272	0.947	−0.013	0.044	0.046	0.002	0.173	0.934
	CLR	−0.023	0.073	0.073	0.006	0.286	0.937	−0.006	0.045	0.047	0.002	0.177	0.939

a Control-to-case ratio, i.e. the number of controls per case in the NCC sub-cohort.

b Estimated standard error.

c Empirical standard error.

d Mean squared error.

e Average length of the 95% confidence intervals.

f Empirical coverage probability of the 95% confidence interval.

**Figure 2:**
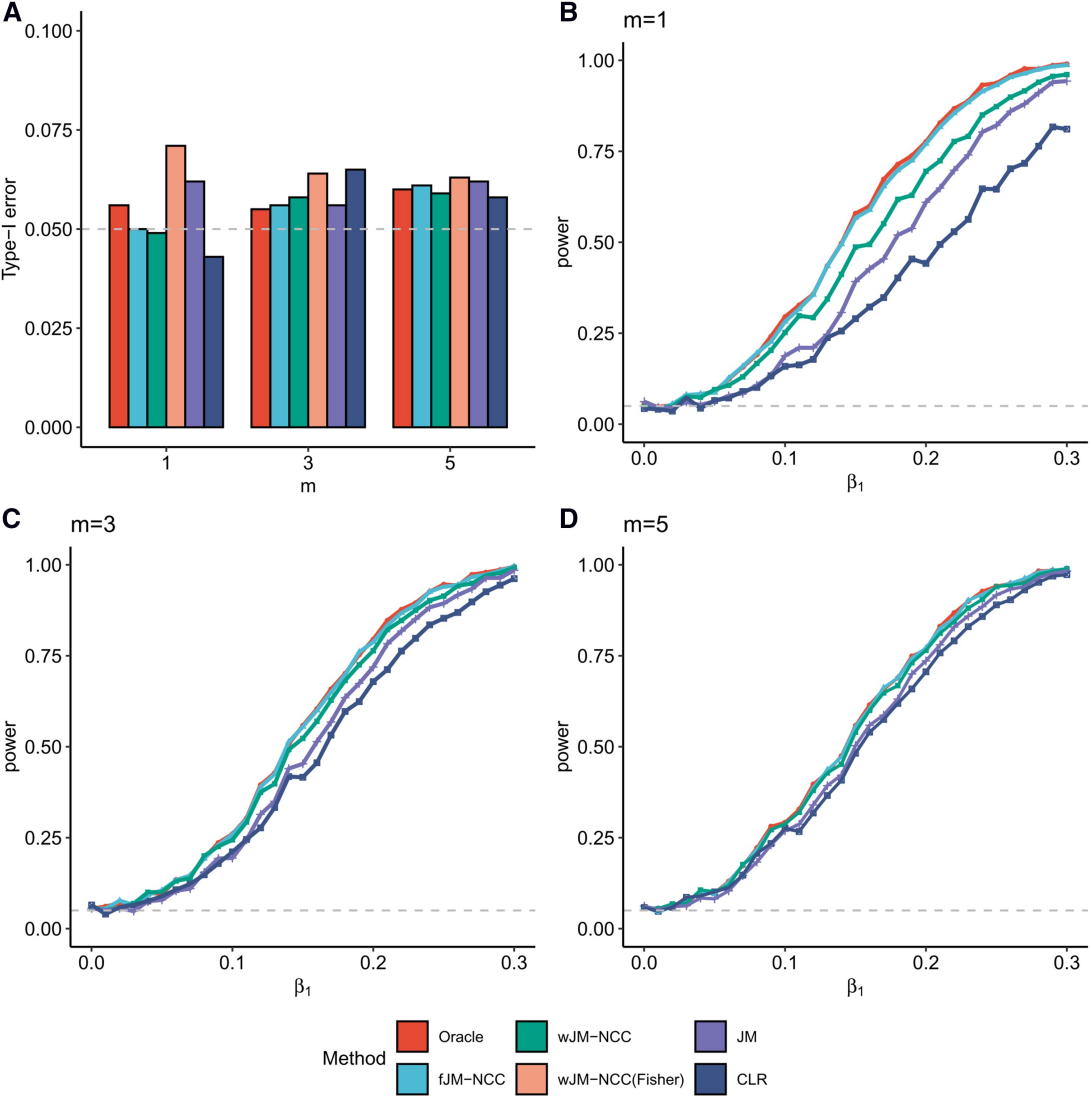
Statistical testing performance of all considered methods for testing $H_{0}:\beta_{1}=0$ under two scenarios in Study 1. (A) Scenario 1: Empirical type-I error rates of all methods for testing $H_{0}:\beta_{1}=0$ with m (control-to-case ratio) ranging from 1 to 5. (B–D) Scenario 2: Statistical power of all methods [excluding wJM-NCC(Fisher) due to inflated type-I error] for $\beta_{1}$ values ranging from 0 to 0.3 in increment of 0.01, with m=1, 3, and 5, and $\beta_{2}$ fixed at 0.1.

Figure 2A shows the empirical type-I error rates for testing $H_{0}:\beta_{1}=0$ under Scenario 1 in Study 1. Results for testing $H_{0}:\beta_{2}=0$ are similar and omitted for brevity. Oracle, fJM-NCC, and wJM-NCC maintain type-I error rates close to the nominal level of 0.05, demonstrating the validity of the proposed methods. wJM-NCC(Fisher) demonstrates inflated type-I error rate due to SE underestimation, and its statistical power is excluded from further comparisons. JM shows elevated type-I error rates when m=1, where the event rate in the fitted NCC sub-cohort is most severely distorted. CLR also shows a higher-than-nominal type-I error rate when m=3.

Figure 2B–D illustrates the statistical power for testing $H_{0}:\beta_{1}=0$ as $\beta_{1}$ increases from 0 to 0.3, and $\beta_{2}$ fixed at 0.1 and m varying from 1 to 5, representing increasing sample sizes of the NCC sub-cohort (Scenario 2, Study 1). Oracle, modeled on the full dataset, serves as the benchmark method and remains its results unchanged as m varies. As expected, Oracle achieves the highest statistical power under all scenarios. fJM-NCC closely follows Oracle, with nearly equivalent power across various values of $\beta_{1}$. The statistical power of wJM-NCC ranks third, but still yields satisfactory results, particularly when compared to the traditional methods JM and CLR. The power loss due to NCC sampling becomes negligible when m=5, as evidenced by nearly identical statistical powers for Oracle, fJM-NCC, and wJM-NCC.

##### 3.1.2.2 Results of study 2

Figure S1, available as supplementary data at *Bioinformatics* online, illustrates the hypothesis testing results for $\beta_{1}$ in Study 2 under the global null ($\beta_{1}=\beta_{2}=0$; panel A) and the alternative ($\beta_{1}=\beta_{2}=0.1$; panel B) across m=1, 3, 5. Consistent with Study 1, Oracle, fJM-NCC, and wJM-NCC maintain type-I error rates close to the nominal 0.05 level, with Oracle achieving the greatest power, followed by fJM-NCC and wJM-NCC. As m increases, the powers of fJM-NCC and wJM-NCC approach that of Oracle. wJM-NCC(Fisher) exhibits inflated type-I error rates. JM shows inflated type-I error at m=1 but approaches the nominal 0.05 as m increases. CLR controls type-I error rates well but suffers from noticeably lower power across all settings. The point and confidence interval estimation results in Study 2 is similar to Study 1 and therefore not shown for brevity.

##### 3.1.2.3 Results of study 3

In Study 3, we further evaluate the hypothesis testing performance of assessed methods with high-dimensional microbiome features that require multiple comparison adjustment. Figure S2, available as supplementary data at *Bioinformatics* online, presents the empirical FDR (A) and TPR (B) for testing overall association effects ($\beta_{1},\;\beta_{2}$) across all methods in Study 3, with m=1, 3, 5. Similar to the results in Study 1, the Oracle, fJM-NCC, wJM-NCC, and CLR maintain FDRs close to the nominal level of 0.05, whereas wJM-NCC(Fisher) and JM exhibit inflated FDRs at m=1. Oracle achieves the highest TPR, followed closely by fJM-NCC and wJM-NCC, both outperforming CLR. As m increases to 5, the TPRs of fJM-NCC, wJM-NCC, and CLR become nearly identical and approach that of the Oracle method.

These results indicate that fJM-NCC performs optimally when longitudinal biomarker data for the NCC sub-cohort and full cohort clinical metadata and survival outcomes are available. Nevertheless, wJM-NCC offers a complementary approach and achieves satisfactory performance, especially when full cohort clinical metadata is not available or difficult to access. Meanwhile, both JM and CLR consistently demonstrate lower statistical power compared to both Oracle method and the proposed methods fJM-NCC and wJM-NCC.

### 3.2 Application to TEDDY microbiome study

Here, we apply the proposed methods, fJM-NCC and wJM-NCC, and competing methods JM and CLR, to the TEDDY microbiome biomarker study (Stewart *et al.* 2018) to investigate the longitudinal microbiome profiles during early human life and their association with the competing appearance of two dominant autoantibodies, IAA (IAA-first) and GADA (GADA-first).

**Figure 3:**
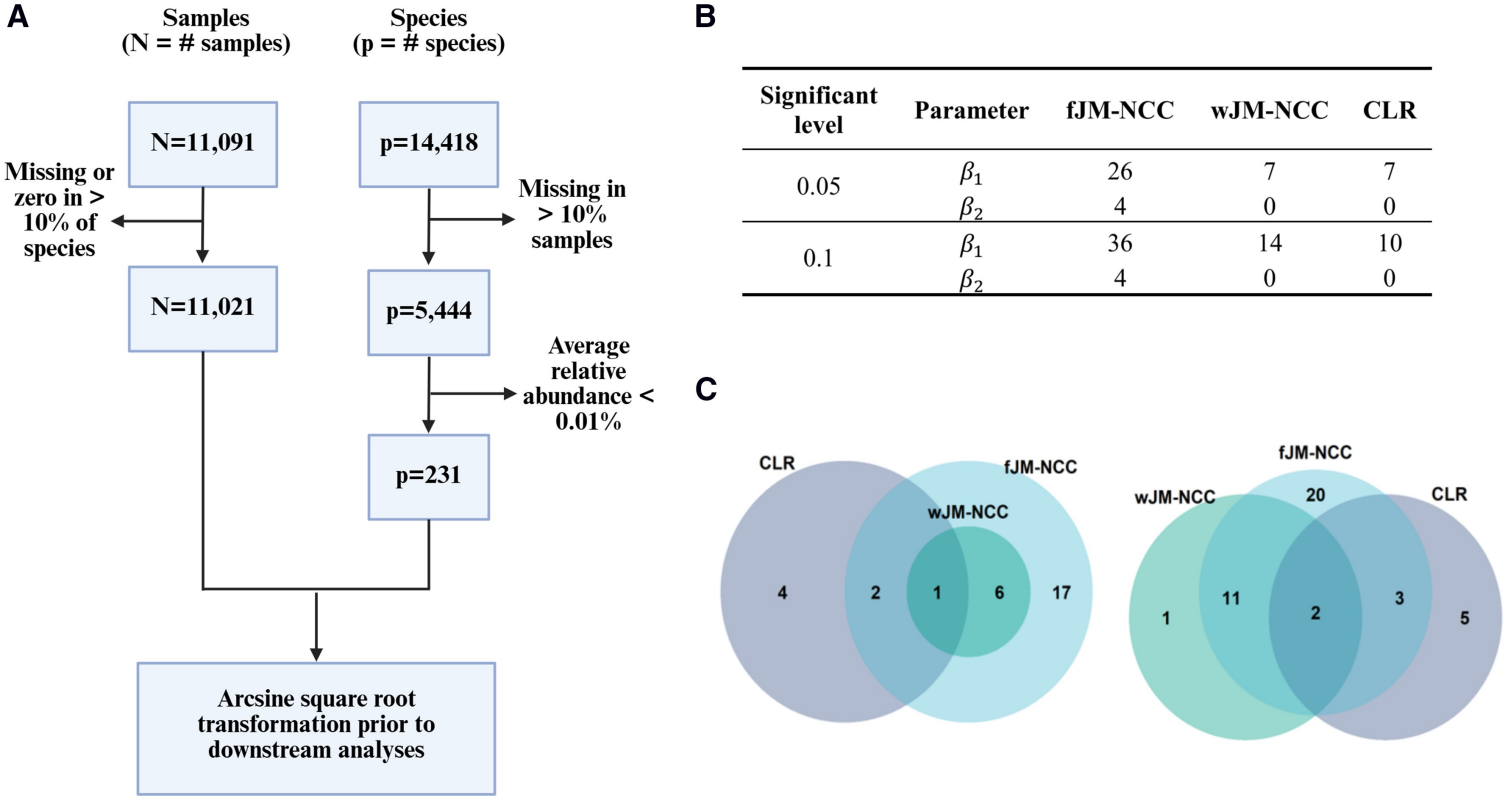
Association analysis of longitudinal microbial abundances with the competing appearance of IAA and GADA using the TEDDY Microbiome dataset. (A) Data filtering steps for shotgun metagenomic sequencing abundance data at the species level from the TEDDY NCC sub-cohort. (B) Number of significant species identified by the assessed methods (fJM-NCC, wJM-NCC, CLR, and JM) at two significant levels, .05 and .1, after Bonferroni correction. JM did not identify any significant species and is therefore excluded from the table. $\beta_{1}$ and $\beta_{2}$ represent the association coefficient between longitudinal species abundances and IAA-first and GADA-first respectively. (C) Venn diagram illustrates the overlap of significant species associated with IAA-first ($\beta_{1}$) as identified by fJM-NCC, wJM-NCC, and CLR at nominal levels of 0.05 (left panel) and 0.1 (right panel), respectively.

After quality control and filtering, six community-level microbiome measurements and 231 microbial species measured across 11 021 samples from 819 subjects from the NCC sub-cohort, and clinical and survival measures of all 8607 participants were retained from downstream association analysis (Fig. 3). Due to its inflated type-I error rate in hypothesis testing, wJM-NCC(Fisher) was excluded from the analysis. Microbial relative abundances were arcsine square root transformed prior to model fitting to improve normality prior to model fitting for all assessed methods. fJM-NCC adopted linear mixed effects model as the longitudinal sub-model throughout the analysis. Details of data preprocessing, imputation, and filtering procedures are provided in Supplementary Materials, Section S6, available as supplementary data at *Bioinformatics* online.

Table 4 summarizes the demographic characteristics of TEDDY cohort, stratified by their case–control status and the first appearing autoantibody. The mean event time for IAA-first was earlier than that for GADA-first, consistent with previous literature (Krischer *et al.* 2015, 2017, Rewers *et al.* 2018). We examined associations between each microbial biomarker (either community-level measurements or species-level arcsine square root transformed relative abundances) and the competing risks of IAA-first and GADA-first by fitting association models for each biomarker and applying multiple comparison adjustments afterwards. Figure S3, available as supplementary data at *Bioinformatics* online, shows the longitudinal trajectories of a representative biomarker (Microbiota Age) in control, IAA-first, and GADA-first groups. The trajectories reveal subject-specific heterogeneity and approximately parallel longitudinal trajectories. Accordingly, in the joint modeling analysis using fJM-NCC, wJM-NCC, and JM, birth mode and sampling time were included as fixed effects in the linear mixed-effects longitudinal sub-model, with a random intercept assumed. The longitudinal microbial trajectory, sex, birth weight, and FDR were included as covariates in the survival sub-model. Since the CLR method cannot handle competing events or time-varying covariates (microbial biomarker herein), mean community-level measures (or the mean relative abundance overtime), birth mode, sex, birth weight, and FDR of IAA-first cases and their controls, as well as those from GADA-first cases and their controls, were included in two separate models to examine microbial association with the appearance of autoantibodies.

**Table 4:** Descriptive characteristics of the TEDDY cohort.^a^

	IAA-first	IAA-control^b^	GADA-first	GADA-control^c^	Controls^d^	Overall
	*N* = 244	*N* = 225	*N* = 113	*N* = 102	*N* = 7923	*N* = 8607
Sex (female)	104 (42.6%)	96 (42.7%)	58 (51.3%)	51 (50.0%)	3947 (49.8%)	4256 (49.4%)
FDR^e^ (yes)	54 (22.1%)	43 (19.1%)	19 (16.8%)	18 (17.6%)	801 (10.1%)	935 (10.9%)
Birth mode						
Caesarian	51 (20.9%)	65 (28.9%)	28 (24.8%)	24 (23.5%)	2134 (26.9%)	2302 (26.7%)
Vaginal	193 (79.1%)	160 (71.1%)	85 (75.2%)	78 (76.5%)	5789 (73.1%)	6305 (73.3%)
Birth weight (kg)	3.58 (0.513)	3.53 (0.593)	3.62 (0.462)	3.52 (0.541)	3.39 (0.578)	3.41 (0.577)
Event time^f^ (year)	1.65 (1.04)	3.99 (0.195)	2.26 (1.32)	4.00 (0.10)	4.00 (0.00)	3.91 (0.501)

a Frequency and proportions, and mean and standard deviation are summarized for categorical and continuous variables, respectively.

b IAA-control: controls matched to IAA-first.

c GADA-control: controls matched to GADA-first.

d Controls: controls unselected into NCC sub-cohort.

e FDR: indicates whether any first-degree relatives in the family have T1D.

f Event time: the time to IA onset (including IAA-first, GADA-first) or censoring time.

Figure S4, available as supplementary data at *Bioinformatics* online, illustrates the piecewise-constant baseline hazards estimated by fJM-NCC for Microbiota Age. IAA-first event shows a higher baseline hazard at earlier time, consistent with its earlier onset pattern. Table 5 presents the estimated parameters ($\beta_{1}$, $\beta_{2}$) and corresponding hypothesis testing p-values from all methods for six community-level microbiome measurements in association with the first appearance of IAA ($\beta_{1})$ and GADA ($\beta_{2})$. Results from fJM-NCC and wJM-NCC are highly consistent, as evidenced by the same direction of parameter estimates and similar levels of statistical significance. The directions of ($\beta_{1}$, $\beta_{2}$) estimated by fJM-NCC and wJM-NCC align well with the interpretation of competing risks. For example, both fJM-NCC and wJM-NCC indicate that lower richness and Shannon diversity (from both 16S rRNA and shotgun metagenomic sequencing data), younger microbiota age, and higher MAZ score are significantly associated with an increased risk for IAA-first. These findings are consistent with the literature suggesting that reduced microbial diversity and delayed microbial maturation are linked to poorer health outcomes and the competing nature of IAA-first and GADA-first (Krischer *et al.* 2017, Stewart *et al.* 2018).

**Table 5:** Estimation results and hypothesis testing *P*-values for the associations between six community-level microbiome measurements and appearance of IAA-first ($\beta_{1}$) and GADA-first ($\beta_{2}$).

Measurements	fJM-NCC	wJM-NCC	JM	CLR
Shotgun metagenomic data				
Richness (# species)	(−0.273, 0.172)^a^(0.001, 0.133)^b^	(−0.169, 0.229)(0.026, 0.043)	(0.196, 0.006)(0.171, 0.978)	(0.528, 0.435)(0.004, 0.139)
Shannon diversity	(−0.413,0.331)(0.002, 0.075)	(−0.315, 0.370)(0.010, 0.051)	(0.349, 0.399)(0.182, 0.300)	(0.471, 0.463)(0.098, 0.267)
16S rRNA sequencing data				
Richness (# OTUs)	(−0.265, 0.253)(<0.001, 0.010)	(−0.245, 0.243)(<0.001, 0.021)	(0.033, 0.390)(0.850, 0.107)	(0.806, 0.638)(<0.001, 0.068)
Shannon diversity	(−0.415, 0.282)(<0.001, 0.071)	(−0.325, 0.322)(0.002, 0.054)	(0.184, −0.074)(0.450, 0.834)	(1.096, 0.931)(0.001, 0.044)
MAZ score	(0.107, −0.087)(<0.001, 0.024)	(0.099, −0.100)(<0.001, 0.043)	(0.001, −0.001)(0.984, 0.992)	(−0.093, −0.043)(0.160, 0.657)
Microbiota age	(−0.035, 0.028)(<0.001, 0.015)	(−0.031, 0.029)(<0.001, 0.019)	(0.030, 0.085)(0.100, <0.001)	(0.157, 0.124)(<0.001, 0.029)

a The point estimates and

b the hypothesis testing *P*-values of ($\beta_{1}$, $\beta_{2}$) for the null hypothesis $H_{0}:\beta_{1}=0$ and $H_{0}:\beta_{2}=0$ respectively.

In contrast, JM provides either identical directional estimates for $\beta_{1}$ and $\beta_{2}$ or results entirely different from those of fJM-NCC and wJM-NCC, suggesting its limitations in NCC designed studies. CLR’s estimates ($\beta_{1}$, $\beta_{2}$) are in the same direction due to the model’s inability to account for the competing risks between IAA-first and GADA-first. The results of CLR indicate that higher microbial diversity, older microbiota age, and lower MAZ score are associated with higher risk of the appearance of either autoantibody, which contradicts existing findings.

The results of the species-level analysis of longitudinal microbial abundances and their association with the competing appearance of IAA-first and GADA-first are summarized in Fig. 3B and C and Table S9, available as supplementary data at *Bioinformatics* online. Figure 3B shows the number of species associated with IAA-first and GADA-first identified by each method at two nominal significant levels (0.05 and 0.1) after Bonferroni correction, while Fig. 3C illustrates the overlap of species identified different methods. Table S9, available as supplementary data at *Bioinformatics* online, provides a full list of the identified species, along with their point estimates and corresponding adjusted p-values for $\left(\beta_{1},\;\beta_{2}\right)$, with Bonferroni adjustments applied separately for $\beta_{1}$ and $\beta_{2}$. For IAA-first, fJM-NCC detected the highest number of significant species, identifying 26 species at the 0.05 level and 36 at the 0.1 level. For GADA-first, it identified four species at both levels. By comparison, wJM-NCC and CLR identified fewer species, with each detecting seven species for IAA-first at the 0.05 level. At the 0.1 level, wJM-NCC identified 14 species, while CLR identified 10. JM failed to identify any significant species. At the 0.05 significance level, all species identified by wJM-NCC were also detected by fJM-NCC, while CLR identified only three overlapping species with fJM-NCC. At the 0.1 level, wJM-NCC identified one species not detected by fJM-NCC, and half of the species identified by CLR overlapped with those detected by fJM-NCC. These findings demonstrate the consistency between methods, and the statistical efficiency of fJM-NCC and wJM-NCC.

We further examined two example species (Table S9, available as supplementary data at *Bioinformatics* online) detected by fJM-NCC and wJM-NCC as significantly associated with autoantibody appearance through cross-referencing findings from existing biomedical literature. *Bacillus cereus* has been reported to enhance the expression of signature genes in Th17 cells, potentially delaying T1D onset (Elhag *et al.* 2020). Consistent with this, the fJM-NCC estimates for *B. cereus* were ${(\beta }_{1},\beta_{2})=$ (−101, −86.2) with adjusted *P*-values (.019, 1), and wJM-NCC estimates were (−100, −86.1) with adjusted *P*-values (.048, 1). In contrast, JM estimates were (−2.5, −0.174) with adjusted *P*-values (1, 1), and CLR estimates were (−1.4, 136.4) with adjusted *P*-values (1, 1), showing significant divergence from fJM-NCC and wJM-NCC. *Bifidobacterium breve*, a dominant gut microbiota species in infancy, has been associated with IA onset in prior studies (Li *et al.* 2022). For this species, fJM-NCC estimates were (1.35, 0.454) with adjusted *P*-values (.075, .392), while wJM-NCC estimates were (0.968, 0.131) with adjusted *P*-values (1, 1). However, JM estimates were (−0.092, 0.821) with adjusted *P*-values (1, 1), and CLR estimates were (−0.84, 0.552) with adjusted *P*-values (1, 1). These examples highlight the strong alignment of fJM-NCC and wJM-NCC with existing biological evidence, while JM and CLR show notable inconsistencies in their inference.

As an augmented analysis, we evaluated type-I error control of assessed methods via a block permutation study of the TEDDY microbiome data. Longitudinal abundances of 231 microbial species from each subject were treated as a single block and randomly permuted across subjects to break associations with competing event outcomes while preserving within-subject correlation. All methods (JM, fJM-NCC, wJM-NCC, and CLR) adequately controlled type-I error rate at α=.05, with empirical type-I error rates of 0.033, 0.054, 0.046, and 0.039 respectively, based on 10 repetitions.

## 4 Discussion

In this article, we propose a novel joint modeling framework specifically designed for NCC studies, aimed at exploring the association between longitudinal biomarker trajectories and competing events. This framework integrates a generalized linear mixed-effects model to capture biomarker dynamics over time and a cause-specific hazard model to link these trajectories to specific competing events. We developed two maximum likelihood estimation approaches, i.e. fJM-NCC and wJM-NCC, to address the unique sampling structure and data characteristics inherent to NCC designs. fJM-NCC leverages data from both the NCC sub-cohort and the full cohort, including survival outcomes and clinical metadata. In contrast, wJM-NCC uses only NCC sub-cohort data and constructs an inverse probability weighting likelihood function to account for the potential selection bias in NCC sampling.

Simulation studies demonstrate the robustness and efficiency of both methods, as evidenced by unbiased parameter estimation and well controlled type-I error rates across various scenarios. The statistical power of fJM-NCC is comparable to that of the Oracle method, which assumes the availability of biomarker data for the full cohort. Although wJM-NCC exhibits slightly lower power than fJM-NCC, its efficiency improves as the number of controls per case (m) increased, gradually approaching Oracle’s performance. In comparison, our proposed methods outperform existing approaches JM, which is unsuitable for NCC designed studies, and conditional logistic regression (CLR), which cannot effectively handle competing events and time-varying covariates. The application of these methods to the TEDDY dataset highlights their practical utility in identifying microbial biomarkers associated with competing events, specifically IAA-first and GADA-first. The current simulation studies focus on survival outcomes generated from a cause-specific hazard model, acknowledging that simulating longitudinal outcomes with competing events under the NCC design is technically challenging. Extending simulations to alternative data-generating mechanisms would provide further insight into the robustness and performance of the proposed methods.

Although this study focused on competing events within the joint modeling framework, it is important to note that the approach can also be applied to single survival outcomes as a special case when the number of competing events (k) is reduced to one. Many diseases, however, progress through multiple intermediate states rather than discrete competing outcomes. For example, T1D development involves transitions from the detection of autoantibodies (any IA phenotype) to the onset of overt disease (Knip *et al.* 2005). Future research could extend our framework to a multi-state setting under the NCC design, enabling the investigation of biomarkers that influence disease progression across different stages. Such an extension would provide deeper insights into the dynamic roles of microbial biomarkers at various stages of disease progression, facilitating the development of more targeted and effective interventions.

## Supplementary Material

btag038_Supplementary_Data

## Data Availability

The TEDDY microbiome 16S rRNA gene sequencing data and shotgun whole-genome sequencing data are publicly available in the NCBI database of Genotypes and Phenotypes (dbGaP) with the primary accession code phs001443.v1.p1, in accordance with the dbGaP controlled-access authorization process. Clinical metadata analyzed during the current study are available in the NIDDK Central Repository at https://www.niddkrepository.org/studies/teddy.
