# Supplementary material for: Joint modeling of longitudinal biomarker and survival outcomes with the presence of competing risk in the nested case–control studies with application to the TEDDY microbiome dataset
Source: Bioinformatics. 2026 Jan 22;42(3):btag038. doi: 10.1093/bioinformatics/btag038 (PMC13005730; doi:10.1093/bioinformatics/btag038)
Supplement: btag038_Supplementary_Data [file btag038_supplementary_data.docx]

Supplementary Materials for

Joint Modeling of Longitudinal Biomarker and Survival Outcomes with the Presence of Competing Risk in Nested Case-Control Studies with Application to the TEDDY Microbiome Dataset

Table of Contents

[S1. Derivation of the numerical approximation of the likelihood function 2](#_Toc218847376)

[S2. Standard error estimation for wJM-NCC 4](#_Toc218847377)

[S3. Simulation setup 4](#_Toc218847378)

[Study 1: Simple longitudinal structure and constant baseline hazards 4](#_Toc218847379)

[Study 2: Complex longitudinal structure and piecewise-constant baseline hazards 5](#_Toc218847380)

[Study 3: Simulating High-dimensional longitudinal microbiome biomarker data using SparseDOSSA2 6](#_Toc218847381)

[S4. Competing methods 7](#_Toc218847382)

[S5. Estimation results of nuisance parameters in Study 1 9](#_Toc218847383)

[S6. Description, data processing, and quality control for TEDDY microbiome study 9](#_Toc218847384)

[S7. Supplementary tables 10](#_Toc218847385)

[S8. Supplementary figures 21](#_Toc218847386)

[**References** 23](#_Toc218847387)

## **S1. Derivation of the numerical approximation of the likelihood function**

Let the time axis be partitioned by $0= v_{0}<v_{1}<\cdots< v_{Q}$. For subject $i$ and competing event $k\in\left\{ 1, \cdots, K \right\}$, the piecewise-constant baseline hazard in the time interval $[v_{q-1}, v_{q})$ is denoted by $\xi_{qk}$.

Condition on the subject-specific random effect $\boldsymbol{b}_{i}$, the cause-specific hazard function for subject $i$ and competing event $k$ is specified as

$$\lambda_{k}\left( t|\boldsymbol{b}_{i} \right)=\xi_{qk}\exp\left( \beta_{k}\cdot g^{-1}\left( \boldsymbol{\gamma}^{T}\boldsymbol{X}_{i}^{\left( 1 \right)}(t)+\boldsymbol{b}_{i}^{T}\boldsymbol{Z}_{i}(t) \right)+\boldsymbol{\alpha}_{k}^{T}\cdot\boldsymbol{X}_{i}^{\left( 2 \right)} \right), v_{q-1}\leq t<v_{q}.$$

The join likelihood function for subject $i$ is given by

$$L_{i}=\left\{ \begin{aligned} &&&\int f\left( T_{i}, \delta_{i}|\boldsymbol{b}_{i},\boldsymbol{X}_{i} \right)f\left( \boldsymbol{Y}_{i}|\boldsymbol{b}_{i},\boldsymbol{X}_{i} \right)f\left( \boldsymbol{b}_{i} \right)d\boldsymbol{b}_{i}, & ifR_{i}=1 \\ &&&\int f\left( T_{i}, \delta_{i}|\boldsymbol{b}_{i},\boldsymbol{X}_{i} \right)f\left( \boldsymbol{b}_{i} \right)d\boldsymbol{b}_{i}, &if R_{i}=0 \end{aligned} \right.,$$

where

$$f\left( T_{i}, \delta_{i}|\boldsymbol{b}_{i},\boldsymbol{X}_{i} \right)=\prod_{k=1}^{K} \left( \lambda_{0k}\left( T_{i} \right)\exp\left( \beta_{k}\cdot g^{-1}\left( \boldsymbol{\gamma}^{T}\boldsymbol{X}_{i}^{\left( 1 \right)}+\boldsymbol{b}_{i}^{T}\boldsymbol{Z}_{i} \right)+\boldsymbol{\alpha}_{k}^{T}\cdot\boldsymbol{X}_{i}^{\left( 2 \right)} \right) \right)^{I\left( \delta_{i}=k \right)}\cdot\exp\left( -\sum_{k=1}^{K} \int_{0}^{T_{i}} \lambda_{0k}\left( s \right)\exp\left( \beta_{k}\cdot g^{-1}\left( \boldsymbol{\gamma}^{T}\boldsymbol{X}_{i}^{\left( 1 \right)}+\boldsymbol{b}_{i}^{T}\boldsymbol{Z}_{i} \right)+\boldsymbol{\alpha}_{k}^{T}\cdot\boldsymbol{X}_{i}^{\left( 2 \right)} \right)ds \right),$$

$$f\left( \boldsymbol{b}_{i} \right)=\frac{1}{\sqrt{2\pi{|\Sigma}_{\theta}|}}\exp\left( -\frac{1}{2}{\boldsymbol{b}_{i}}^{T}{\Sigma_{\theta}}^{-1}\boldsymbol{b}_{i} \right), \mathrm{and}$$

$$f\left( \boldsymbol{Y}_{i}|\boldsymbol{b}_{i},\boldsymbol{X}_{i} \right)=\prod_{j=1}^{n_{i}} \exp\left( \frac{Y_{ij}\cdot\eta_{ij}-B\left( \eta_{ij} \right)}{a_{ij}(\tau)}+C(Y_{ij}, \tau) \right),$$

where $\eta_{ij}$ (the nature parameter) is associated with the conditional mean $\mu_{ij}$, $B(\cdot)$ and C$(\cdot)$ are known distribution-specific functions (Jiang 2007).
For subject $i$ and competing event $k$, the conditional density of the survival data given the random effects can been written as

$$f\left( T_{i}, \delta_{i}=k|\boldsymbol{b}_{i},\boldsymbol{X}_{i} \right)=\lambda_{k}\left( T_{i}|\boldsymbol{b}_{i} \right)\exp\left\{ -\sum_{k=1}^{K} \int_{0}^{T_{i}} \lambda_{k}\left( s|\boldsymbol{b}_{i} \right)ds \right\}, k=1,\cdots, K,$$

and for censored observation ($\delta_{i}=0$):

$$f\left( T_{i}, \delta_{i}=0|\boldsymbol{b}_{i},\boldsymbol{X}_{i} \right)=\exp\left\{ -\sum_{k=1}^{K} \int_{0}^{T_{i}} \lambda_{k}\left( s|\boldsymbol{b}_{i} \right)ds \right\}.$$

The corresponding log-density for an observed event of cause $k$ is

$$\log f\left( T_{i}, \delta_{i}=k|\boldsymbol{b}_{i},\boldsymbol{X}_{i} \right)=\log\lambda_{k}\left( T_{i}|\boldsymbol{b}_{i} \right)-\sum_{k=1}^{K} \int_{0}^{T_{i}} \lambda_{k}\left( s|\boldsymbol{b}_{i} \right)ds,$$

where $\log\lambda_{k}\left( T_{i}|\boldsymbol{b}_{i} \right)=\log\xi_{Qk}+\beta_{k}\cdot g^{-1}\left( \boldsymbol{\gamma}^{T}\boldsymbol{X}_{i}^{\left( 1 \right)}(T_{i})+\boldsymbol{b}_{i}^{T}\boldsymbol{Z}_{i}(T_{i}) \right)+\boldsymbol{\alpha}_{k}^{T}\cdot\boldsymbol{X}_{i}^{\left( 2 \right)}$.

The integral over the random effects is approximated using multidimensional Gauss-Hermite (GH) quadrature:

$$\int h\left( b \right)\varphi\left( b;0, \Sigma_{\theta} \right)db\approx\sum_{m=1}^{M} w_{m}h\left( b_{m} \right),$$

where $b_{m}$ and $w_{m}$ are the GH nodes and weights after a Cholesky-based linear transformation based on the covariance matrix $\Sigma_{\theta}$, such that the approximation holds for integrals with respect to $N(0, \Sigma_{\theta})$.

Thus, for subjects with longitudinal measurements ($R_{i}=1$):

$$L_{i}\approx\sum_{m=1}^{M} w_{m}f\left( T_{i}, \delta_{i}|\boldsymbol{b}_{m},\boldsymbol{X}_{i} \right)f\left( \boldsymbol{Y}_{i}|\boldsymbol{b}_{m},\boldsymbol{X}_{i} \right).$$

For $R_{i}=0$, $f\left( \boldsymbol{Y}_{i}|\boldsymbol{b}_{m},\boldsymbol{X}_{i} \right)=1$.

For numerical stability, the log-likelihood is computed using the log-sum-exp transformation:

$$\log L_{i}\approx\log\sum_{m=1}^{M} \exp\left( \log w_{m}+\log f\left( T_{i}, \delta_{i}|\boldsymbol{b}_{m},\boldsymbol{X}_{i} \right)+\log f\left( \boldsymbol{Y}_{i}|\boldsymbol{b}_{m},\boldsymbol{X}_{i} \right) \right).$$

The cumulative hazard term $\int_{0}^{T_{i}} \lambda_{k}\left( s|\boldsymbol{b}_{m} \right)ds$ is evaluated numerically because the longitudinal predictor $g^{-1}\left( \boldsymbol{\gamma}^{T}\boldsymbol{X}_{i}^{\left( 1 \right)}(s)+\boldsymbol{b}_{m}^{T}\boldsymbol{Z}_{i}(s) \right)$ is time-dependent and has no closed form.

The integration is partitioned by the baseline hazard intervals:

$$\int_{0}^{T_{i}} \lambda_{k}\left( s|\boldsymbol{b}_{m} \right)ds=\sum_{q:U_{iq}>L_{iq}}^{Q} \int_{L_{iq}}^{U_{iq}} \xi_{qk}\exp\left( \beta_{k}\cdot g^{-1}\left( \boldsymbol{\gamma}^{T}\boldsymbol{X}_{i}^{\left( 1 \right)}(s)+\boldsymbol{b}_{m}^{T}\boldsymbol{Z}_{i}(s) \right)+\boldsymbol{\alpha}_{k}^{T}\cdot\boldsymbol{X}_{i}^{\left( 2 \right)} \right)ds,$$

where $U_{iq}=\min(v_{q}, T_{i})$ and $L_{iq}=\max(0, v_{q-1})$.

Each integral on $[L_{iq}, U_{iq}]$ is approximated using $R$-point Gauss-Kronrod (GK) quadrature:

$$\int_{L_{iq}}^{U_{iq}} (\cdot)ds\approx\sum_{r=1}^{R} w_{r}\left( \frac{U_{iq}-L_{iq}}{2} \right)\xi_{qk}\exp\left\{ \beta_{k}\cdot g^{-1}\left( {\boldsymbol{\gamma}^{T}\boldsymbol{X}}_{i}^{\left( 1 \right)}\left( u_{qr} \right)+\boldsymbol{b}_{m}^{T}\boldsymbol{Z}_{i}\left( u_{qr} \right) \right)+\boldsymbol{\alpha}_{k}^{T}\cdot\boldsymbol{X}_{i}^{\left( 2 \right)} \right\}$$

where $u_{qr}=\left( \frac{U_{iq}-L_{iq}}{2} \right) s_{r}+\left( \frac{U_{iq}+L_{iq}}{2} \right)$, and $(s_{r}, w_{r})$ are the standard GK quadrature nodes and weights. In our numerical studies, we used 7 GK quadrature points and 15 GH quadrature points per dimension for the approximation.

Based on the above derivation, we can obtain the numerical approximation of the log-likelihood functions for both fJM-NCC and wJM-NCC.

## **S2. Standard error estimation for wJM-NCC**

In wJM-NCC, direct application of the Fisher information matrix from the weighted log-likelihood tends to underestimate the standard errors. Therefore, we employed the sandwich covariance estimator to achieve more robust variance estimation:

$$\mathrm{Cov}\left( \boldsymbol{\phi} \right)=I^{-1}\left( \boldsymbol{\phi} \right)\Sigma\left( \boldsymbol{\Phi} \right)I^{-1}\left( \boldsymbol{\phi} \right),$$

where $I\left( \boldsymbol{\phi} \right)$ and $\Sigma\left( \boldsymbol{\phi} \right)$ denote the Fisher information matrix and the covariance matrix of the score function, respectively. These quantities can be consistently estimated by their empirical counterparts as follows:

$$\hat{I}\left( {\hat{\boldsymbol{\phi}}}_{\mathrm{wt}} \right)=-\frac{1}{N_{1}}\frac{\partial^{2}}{\partial\boldsymbol{\phi}\partial\boldsymbol{\phi}^{T}}l_{\mathrm{wt}}\left( {\hat{\boldsymbol{\phi}}}_{\mathrm{wt}} \right),$$

$$\hat{\Sigma}\left( {\hat{\boldsymbol{\phi}}}_{\mathrm{wt}} \right)=\frac{1}{N_{1}}\sum_{i, R_{i}=1} \left[ \frac{\partial l_{wt, i}\left( {\hat{\boldsymbol{\phi}}}_{\mathrm{wt}} \right)}{\partial\boldsymbol{\phi}}-\frac{1}{N_{1}}\sum_{i, R_{i}=1} \frac{\partial l_{wt, i}\left( {\hat{\boldsymbol{\phi}}}_{\mathrm{wt}} \right)}{\partial\boldsymbol{\phi}} \right]^{\otimes^{2}},$$

where ${\hat{\boldsymbol{\phi}}}_{\mathrm{wt}}$ is the MLE of $\boldsymbol{\phi}$ obtained by the wJM-NCC approach, and $\boldsymbol{a}^{\otimes^{2}}=\boldsymbol{a}\boldsymbol{a}^{T}$ for any vector $\boldsymbol{a}$.

## **S3. Simulation setup**

We conducted three simulation studies to evaluate the performance of the proposed methods under different longitudinal and survival data structures, as well as in microbiome-inspired settings.

### **Study 1: Simple longitudinal structure and constant baseline hazards**

Longitudinal biomarker measurements were generated using a linear mixed-effects model with a fixed slope and random intercept:

$$Y_{ij}=\gamma\cdot t_{ij}{+ b}_{0i}+\epsilon_{ij},$$

where each subject $i$had five repeated measurements $(j=0,1,\cdots,4)$ recorded at times $t_{ij}$=0, 0.1, 0.2, 0.3, 0.4. The fixed slope was set to $\gamma=0.1,$the random intercept was drawn from $b_{0i}\sim N\left( 0, \theta^{2}=2 \right)$, and the residual errors followed $\epsilon_{ij}\sim N(0,\sigma^{2}=1)$.

To simulate competing event times, we considered a dichotomous covariate $X_{i}^{(2)}\sim Bernoulli(0.5$), representing gender, with a coefficient $\alpha=-0.2$ in the cause-specific hazard model. For subject $i$ and cause $k \in\{1, 2\}$, the cause-specific hazard was defined as

$$\lambda_{k}\left（ t \right）=\lambda_{0k}\exp\left( \beta_{k}\cdot\left( \gamma\cdot t_{ij}+b_{0i} \right)+\alpha\cdot X_{i}^{(2)} \right),$$

where two competing events ($k=1, 2)$ were considered with constant baseline hazards $\log\left( \lambda_{01} \right)=-5$ and $\log\left( \lambda_{02} \right)=-4$ , respectively. Event times were simulated using inverse transform sampling based on these hazard functions, and the observed event time was defined as the minimum of the two cause-specific event times or censoring time.

- **Simulation scenarios for study 1**

We considered two scenarios to evaluate the performance of the proposed methods in the statistical inference of $\beta_{1}$ and $\beta_{2}$:

- - **Scenario 1:** Global Null Hypothesis: ${\beta_{1}=\beta}_{2}=0,$ indicating no association between the biomarker trajectory and either event.
  - **Scenario 2:** Alternative Hypothesis: we fixed $\beta_{2}=0.1$ and varied $\beta_{1}$ from 0 (no association with event 1, no competing) to 0.3. This allows us to examine performance under alternative hypotheses with varying effect sizes for event 1.

### **Study 2: Complex longitudinal structure and piecewise-constant baseline hazards**

This simulation study introduced additional heterogeneity by incorporating both random intercepts and random slopes to allow more complex subject-specific longitudinal trajectories.

For each subject $i$, the random effects $(b_{0i},b_{1i})$ were generated from a mean-zero bivariate normal distribution with $Var\left( b_{0i} \right)=1.2, Var\left( b_{1i} \right)=1.5$, and $Corr\left( b_{0i}, b_{1i} \right)=0.5$. The longitudinal biomarker measurements were then modeled as

$$Y_{ij}=\gamma_{0}+\gamma\cdot t_{ij}+b_{0i}+b_{1i}\cdot t_{ij}+\epsilon_{ij},$$

with the same measurement times $t_{ij}$=0, 0.1, 0.2, 0.3, 0.4 and $\epsilon_{ij}\sim N(0,\sigma^{2}=1)$. The fixed intercept and slope were set to $\gamma_{0}=0.2$ and $\gamma=0.8$.

The true baseline hazards for the competing events were specified as piecewise-constant functions. For event 1 and event 2, the log-transformed baseline hazards were given by:

$\log\left( \lambda_{01} \right)=\left( -5, -4, -2 \right)$, $\log\left( \lambda_{02} \right)=\left( -4, -3, -2 \right)$,

corresponding to time intervals $\left[ 0, 0.2 \right), \left[ 0.2, 0.4 \right), [0.4, \infty)$, respectively. The covariate $X_{i}^{(2)}$ was generated in the same way as in Study 1, with an effect size $\alpha=0.3$. Other simulation parameters were consistent with those in Study 1.

We considered global null (${\beta_{1}=\beta}_{2}=0$) and alternative (${\beta_{1}=\beta}_{2}=0.1$) to evaluate the performance of the proposed methods in the statistical inference of $\beta_{1}$ and $\beta_{2}$.

### **Study 3: Simulating High-dimensional longitudinal microbiome biomarker data using SparseDOSSA2**

In this study, we aimed to mimic real longitudinal microbiome biomarker data using an existing microbiome data simulator. SparseDOSSA2 (Ma 2021) was adopted to generate baseline microbiome profiles for 8,000 simulated subjects, producing 100 taxonomic biomarker features without any association with metadata, based on the “Stool” template of the R package. Based on these baseline profiles, we then simulated longitudinal biomarker measurements at five time points ($t_{ij}$= 0, 0.1, 0.2, 0.3, 0.4) for each feature using a zero-inflated log-normal (ZILN) model. For each feature, two components were specified: 1) Presence probability (non-zero): set to the taxon’s prevalence rate at baseline; 2) non-zero abundance: modeled as

$$\log Y_{ij}\sim N\left( \mu_{ij},\varphi\right),$$

where $\mu_{ij}=\mu+0.8\cdot t_{ij}+b_{0i}+\epsilon_{ij}$, $b_{0i}\sim N(0, {1.2}^{2})$, and $\epsilon_{ij}\sim N(0, 1)$. Here, $\mu$ and $\varphi$ represent the empirical mean and variance of the non-zero log-transformed abundances for the corresponding feature at baseline. The cause-specific baseline hazards were defined as piecewise-constant functions, with $log(\lambda_{01})=\left( -6, -5, -6 \right)$, $log(\lambda_{02})=\left( -6, -4, -4 \right)$, corresponding to time intervals$\left[ 0, 0.2 \right)$, $\left[ 0.2, 0.4 \right)$, $[0.4, \infty)$, respectively.

The covariate $X_{i}^{(2)}$ was generated in the same way as in Study 1, with an effect size $\alpha=0.3$. Other parameters, including the number of cases and selected controls in NCC sub-cohort and the censoring time schemes, were kept consistent with those in Study 1 and 2.

To introduce true associations, we randomly selected 10% of features with prevalence greater than 0.1 and assigned effect sizes $\beta_{1}=0.5$ and $\beta_{2}=-0.5$ for the two competing risks, respectively.

Microbiome abundance data were log-transformed prior to model fitting for all assessed methods. Linear mixed-effects model was employed as the longitudinal sub-model for fJM-NCC and wJM-NCC.

For Study 1, Study 2, and Study 3, the full cohort consisted of $N=8 ,000$ subjects. The first 400 subjects experiencing either event 1 or event 2 were selected to form the cases in the NCC sub-cohort. Two censoring schemes were applied: 1) Random censoring: Censoring times were drawn from a uniform distribution to mimic general right-censoring; 2) Administrative censoring: The censoring time was set to the maximum event time among the first 400 observed events to ensure exactly 400 cases.

For each case, we randomly selected $m = 1, 3, or 5$controls from the corresponding risk set, matched by gender $X_{i}^{(2)}$, to construct the NCC sub-cohort. Covariates, longitudinal and survival outcome data were then extracted for each selected individual to form the NCC dataset, resulting in sub-cohort sizes of $10\%$($m$ =1), 20% ($m$ =3), and 30% ($m$ =5) of the full cohort size.

For Studies 1 and 2, bias and mean squared error (MSE) of point estimates, and the corresponding mean standard errors (SE) and empirical standard errors (ESE) are employed to evaluate the point estimates of model parameters. Confidence Intervals (CI) estimates were assessed using the average length of 95% confidence intervals (CI-L) and empirical coverage probability (ECP). We further evaluated Type-I error rates and statistical power for hypothesis tests. A total of 1000 repetitions were conducted to evaluate the parameter estimates and hypothesis testing performance for each parameter combination, with a nominal significance level of 0.05.

For Study 3, to evaluate the performance of fJM-NCC and wJM-NCC, we calculated empirical discovery rate (FDR) and true positive rate (TPR) across different methods for testing the association effects $(\beta_{1}, \beta_{2})$ at FDR threshold of 0.05. Benjamini-Hochberg (BH) procedure was applied separately to the raw p-values of $\beta_{1}$ and $\beta_{2}$ to obtain their respective adjusted p-values. A total of 100 repetitions were performed.

## **S4. Competing methods**

**Oracle method:** In the simulation studies, covariates, longitudinal, and survival outcomes data were generated for all individuals in the full cohort. As a benchmark, we used a joint modeling approach with competing events modeled that assumes the full cohort data, i.e., covariates, longitudinal and survival outcomes are available for all subjects. This approach, denoted as the Oracle method, uses the log-likelihood function $l_{\mathrm{oracle}}\left( \boldsymbol{\phi} \right)=\sum_{i=1}^{N} \log\left( \int f\left( T_{i}, \delta_{i}|\boldsymbol{b}_{i},\boldsymbol{X}_{i} \right)f\left( \boldsymbol{Y}_{i}|\boldsymbol{b}_{i},\boldsymbol{X}_{i} \right)f\left( \boldsymbol{b}_{i} \right)d\boldsymbol{b}_{i} \right)$ for parameter estimation and hypothesis testing, where the pdfs are defined in Section S1. The Oracle method serves as a baseline to highlight the benefits of our methods designed for NCC sampling. While the Oracle method relies on the full cohort data, our NCC-based methods reduce sample size requirements for biomarker studies and achieve minimal efficiency loss compared to the Oracle method in parameter estimation and hypothesis testing.

**wJM-NCC(Fisher) method:** To evaluate standard error estimation calibration in our proposed wJM-NCC method, we also included the weighted likelihood approach used in wJM-NCC, but with standard error estimates derived from the Fisher’s information matrix. We denote this approach as wJM-NCC(Fisher). Note that the point estimates from both wJM-NCC and wJM-NCC(Fisher) are the same; the only difference lies in the standard error estimates for the studied parameters. This comparison highlights the underestimation of standard errors in the Fisher method, which the sandwich estimator in wJM-NCC avoids.

**JM method:** Additionally, we included the classical joint modeling approach for longitudinal outcomes and competing events, as proposed by Williamson (2008). This approach assumes a Gaussian linear model for longitudinal outcomes, and a semi-parametric cause-specific hazard model for the competing survival outcomes. Implemented in the R package JM (Rizopoulos 2010), the method can be executed using the function jointModel(lmeObject, survObject, timeVar="obsTime", method="spline-PH-GH", CompRisk = TRUE, interFact = list(value = ~ causeEvent, data = cc.data.long)), where cc.data.long is the competing risks long format data of NCC sub-cohort obtained using function crLong(). The matching variable $X_{i}^{(2)}$ is included as a covariate, similar to Oracle, fJM-NCC, wJM-NCC, and wJM-NCC(Fisher). We refer to this method as JM in this article. Since JM requires both longitudinal and survival data for each individual included in the model, it can only be applied to the NCC sub-cohort, as longitudinal measurements are unavailable for individuals not selected into the NCC sub-cohort (i.e., where $R_{i}=0$).

**CLR method:** Conditional logistic regression (CLR) is a standard approach for analyzing matched case-control data to study the relationship between risk factors and a dichotomous outcome with strata (Gail 1981). Given that NCC sampling with matching can be considered a matched case-control design -- and has been applied to analyze TEDDY data (Norris 2018, Lee 2020, Auchtung 2022) -- CLR was included in our comparison with some necessary adaptations. First, because CLR cannot accommodate time-varying covariates (e.g., longitudinal biomarker measurements), we included each individual’s average biomarker measurement as a covariate. Second, cases and their matched controls were analyzed for each type of competing event to estimate parameters specific to that event. Thus, CLR results reflect the association between average biomarker values and each individual event, rather than a direct inference on competing risks.

## **S5. Estimation results of nuisance parameters in Study 1**

**Tables S4-S8** present estimates for other parameters in Study 1, including the fixed slope $\gamma$, the standard deviation (log) of random intercept $\log(\theta)$, the standard deviation (log) of random error $\log\left( \sigma\right)$ in the longitudinal sub-model, and the fixed effect $\alpha$ of the covariate in the survival sub-model. CLR is excluded as it does not provide estimates for these parameters. JM does not provide SE estimates for $\log(\theta)$ and $log(\sigma)$ and therefore performance of JM for these parameters are not reported. Oracle, fJM-NCC, and wJM-NCC, as expected, yield minimal biased point estimates for all parameters. In contrast, JM shows bias for $\log(\theta)$, $log(\sigma)$, and $\alpha$ due to that it treats NCC sub-cohort as the full cohort. SE estimates from Oracle and fJM-NCC consistently align closely with their corresponding ESEs, while wJM-NCC provides consistent SE estimates for the longitudinal data generation parameters ($\gamma$, $log(\theta)$, and $\log\left( \sigma\right)$) but slightly conservative SEs for $\alpha$. wJM-NCC(Fisher) underestimate SEs for for $\gamma$, $\log(\theta)$, and $\log\left( \sigma\right)$. JM exhibits a consistent SE estimate for $\gamma$ but a significant conservative SE estimate for $\alpha$. In summary, these results indicate that JM and CLR are not suitable for analyzing NCC designed longitudinal biomarker studies with competing events.

## **S6. Description, data processing, and quality control for TEDDY microbiome study**

TEDDY study is a prospective birth cohort that has followed 8, 676 newborns over 15 years to study the development of persistent confirmed islet autoimmunity (IA, the pre-clinical phase of T1D) and the diagnosis of T1D. TEDDY aims to evaluate host genetics, gene expression, dietary biomarkers, metabolomics, microbiome, and virome in association to T1D. To efficiently study these biomarkers, TEDDY conducted the biomarker study in a nested case-control (NCC) sub-cohort (Lee 2014, Rundle 2012, Rundle 2005). Specifically, subjects with persistent IA and T1D are included as cases, while autoantibody-negative subjects at the case’s event age, matched by clinical center, sex, and family history of T1D are selected as controls to form the NCC sub-cohort. Only biospecimen from the NCC sub-cohort were processed to evaluate the corresponding biomarkers in relation to IA/T1D onset.

For this secondary data analysis, clinical metadata accessible to our research group were extracted from the full TEDDY cohort (N = 8,607), including sex, birth mode, birth weight, and the status of having a first-degree relative with T1D (FDR). Missing data were imputed using a binormal distribution for birth mode and FDR, and a normal distribution for birth weight, with parameters estimated from subjects with complete data.

Microbiome data included community-level measurements and temporal shotgun metagenomic species-level taxa abundances from the NCC-sub cohort of 357 children who developed IAA first (N= 244) or GADA first (N = 113), along with their matched controls (N = 327). Community-level microbiome measurements include four alpha diversity indices, i.e., microbial richness (number of OTUs/species) and Shannon’s diversity indices calculated from both 16S rRNA sequencing and shotgun metagenomic sequencing data, and two microbiome maturation indices: Microbiota-by-age Z-scores (MAZ) and microbiota age derived from 16S rRNA sequencing data (Stewart 2018, Subramanian 2014).

Data filtering steps are performed at both the microbiome sample and species level for the shotgun metagenomic taxa abundances. Microbiome samples with >10% missingness or zero abundances across all species were excluded, and species detected in fewer than 10% of samples or with an average relative abundance <0.01% were removed (Figure 3A). After these quality control steps, the relative abundances (arcsine square root transformed) of 231 species from 11,021 samples (819 subjects) are retained for downstream association analysis.

## **S7. Supplementary tables**

**Table S1**: Performance of all methods for point and 95% confidence interval estimation of $\beta_{1}$ and $\beta_{2}$ under **Scenario 2** ($\beta_{1}=0$ and $\beta_{2}=0.1$) in Study 1.

|  |  | $\beta_{1}$ | | | | | |  | $\beta_{2}$ | | | | | |
| --- | --- | --- | --- | --- | --- | --- | --- | --- | --- | --- | --- | --- | --- | --- |
| **m^1^** | **Method** | **Bias** | **SE^2^** | **ESE^3^** | **MSE^4^** | **CI-L^5^** | **ECP^6^** |  | **Bias** | **SE** | **ESE** | **MSE** | **CI-L** | **ECP** |
| 1 | Oracle | 0.001 | 0.073 | 0.074 | 0.006 | 0.287 | 0.944 |  | 0.000 | 0.044 | 0.043 | 0.002 | 0.171 | 0.946 |
|  | fJM-NCC | 0.001 | 0.075 | 0.077 | 0.006 | 0.294 | 0.950 |  | 0.002 | 0.046 | 0.046 | 0.002 | 0.182 | 0.946 |
|  | wJM-NCC | 0.003 | 0.082 | 0.083 | 0.007 | 0.322 | 0.950 |  | 0.003 | 0.058 | 0.059 | 0.004 | 0.227 | 0.946 |
|  | wJM-NCC(Fisher) | 0.003 | 0.073 | 0.083 | 0.007 | 0.288 | 0.929 |  | 0.003 | 0.044 | 0.059 | 0.004 | 0.172 | 0.850 |
|  | JM | -0.019 | 0.073 | 0.073 | 0.006 | 0.285 | 0.938 |  | -0.022 | 0.044 | 0.045 | 0.002 | 0.171 | 0.914 |
|  | CLR | 0.003 | 0.098 | 0.101 | 0.010 | 0.384 | 0.956 |  | -0.009 | 0.058 | 0.058 | 0.003 | 0.228 | 0.949 |
| 3 | Oracle | 0.002 | 0.073 | 0.075 | 0.006 | 0.287 | 0.945 |  | 0.001 | 0.044 | 0.044 | 0.002 | 0.171 | 0.946 |
|  | fJM-NCC | 0.002 | 0.074 | 0.078 | 0.006 | 0.291 | 0.945 |  | 0.002 | 0.045 | 0.046 | 0.002 | 0.178 | 0.957 |
|  | wJM-NCC | 0.001 | 0.076 | 0.079 | 0.006 | 0.298 | 0.943 |  | 0.000 | 0.049 | 0.050 | 0.002 | 0.190 | 0.946 |
|  | wJM-NCC(Fisher) | 0.001 | 0.073 | 0.079 | 0.006 | 0.287 | 0.937 |  | 0.000 | 0.044 | 0.050 | 0.002 | 0.172 | 0.921 |
|  | JM | -0.008 | 0.073 | 0.075 | 0.006 | 0.285 | 0.944 |  | -0.011 | 0.044 | 0.044 | 0.002 | 0.171 | 0.941 |
|  | CLR | 0.004 | 0.078 | 0.081 | 0.007 | 0.306 | 0.935 |  | -0.008 | 0.047 | 0.048 | 0.002 | 0.184 | 0.945 |
| 5 | Oracle | -0.005 | 0.073 | 0.074 | 0.005 | 0.286 | 0.940 |  | 0.001 | 0.044 | 0.043 | 0.002 | 0.171 | 0.953 |
|  | fJM-NCC | -0.005 | 0.074 | 0.075 | 0.006 | 0.290 | 0.939 |  | 0.002 | 0.045 | 0.045 | 0.002 | 0.176 | 0.955 |
|  | wJM-NCC | -0.005 | 0.074 | 0.076 | 0.006 | 0.291 | 0.941 |  | 0.001 | 0.046 | 0.046 | 0.002 | 0.182 | 0.945 |
|  | wJM-NCC(Fisher) | -0.005 | 0.073 | 0.076 | 0.006 | 0.286 | 0.936 |  | 0.001 | 0.044 | 0.046 | 0.002 | 0.172 | 0.927 |
|  | JM | -0.010 | 0.072 | 0.074 | 0.006 | 0.284 | 0.938 |  | -0.007 | 0.043 | 0.043 | 0.002 | 0.170 | 0.947 |
|  | CLR | -0.003 | 0.074 | 0.075 | 0.006 | 0.289 | 0.942 |  | -0.008 | 0.044 | 0.045 | 0.002 | 0.174 | 0.945 |

1. Control-to-case ratio, i.e., the number of controls per case in the NCC sub-cohort

2. Estimated standard error

3. Empirical standard error

4. Mean squared error

5. Average length of the 95% confidence intervals

6. Empirical coverage probability of the 95% confidence interval

**Table S2:** Performance of all methods for point and 95% confidence interval estimation of $\beta_{1}$ and $\beta_{2}$ under **Scenario 2** ($\beta_{1}=0.1$ and $\beta_{2}=0.1$) in Study 1.

|  |  | $\beta_{1}$ | | | | | |  | $\beta_{2}$ | | | | | |
| --- | --- | --- | --- | --- | --- | --- | --- | --- | --- | --- | --- | --- | --- | --- |
| **m^1^** | **Method** | **Bias** | **SE^2^** | **ESE^3^** | **MSE^4^** | **CI-L^5^** | **ECP^6^** |  | **Bias** | **SE** | **ESE** | **MSE** | **CI-L** | **ECP** |
| 1 | Oracle | 0.002 | 0.072 | 0.073 | 0.005 | 0.284 | 0.943 |  | 0.000 | 0.044 | 0.044 | 0.002 | 0.172 | 0.953 |
|  | fJM-NCC | 0.003 | 0.074 | 0.077 | 0.006 | 0.292 | 0.940 |  | 0.001 | 0.046 | 0.048 | 0.002 | 0.182 | 0.939 |
|  | wJM-NCC | 0.004 | 0.082 | 0.082 | 0.007 | 0.320 | 0.953 |  | 0.002 | 0.058 | 0.058 | 0.003 | 0.227 | 0.957 |
|  | wJM-NCC(Fisher) | 0.004 | 0.073 | 0.082 | 0.007 | 0.284 | 0.911 |  | 0.002 | 0.044 | 0.058 | 0.003 | 0.172 | 0.864 |
|  | JM | -0.026 | 0.072 | 0.083 | 0.008 | 0.282 | 0.933 |  | -0.030 | 0.044 | 0.045 | 0.003 | 0.171 | 0.899 |
|  | CLR | -0.002 | 0.098 | 0.101 | 0.010 | 0.383 | 0.951 |  | -0.009 | 0.058 | 0.059 | 0.004 | 0.228 | 0.947 |
| 3 | Oracle | -0.003 | 0.072 | 0.073 | 0.005 | 0.284 | 0.946 |  | -0.003 | 0.044 | 0.043 | 0.002 | 0.172 | 0.961 |
|  | fJM-NCC | -0.002 | 0.074 | 0.074 | 0.005 | 0.288 | 0.946 |  | -0.002 | 0.045 | 0.044 | 0.002 | 0.178 | 0.959 |
|  | wJM-NCC | -0.003 | 0.075 | 0.075 | 0.006 | 0.294 | 0.943 |  | -0.002 | 0.049 | 0.049 | 0.002 | 0.191 | 0.946 |
|  | wJM-NCC(Fisher) | -0.003 | 0.072 | 0.075 | 0.006 | 0.284 | 0.939 |  | -0.002 | 0.044 | 0.049 | 0.002 | 0.172 | 0.920 |
|  | JM | -0.019 | 0.072 | 0.070 | 0.005 | 0.282 | 0.952 |  | -0.016 | 0.044 | 0.043 | 0.002 | 0.171 | 0.937 |
|  | CLR | -0.007 | 0.078 | 0.079 | 0.006 | 0.305 | 0.945 |  | -0.011 | 0.047 | 0.046 | 0.002 | 0.184 | 0.951 |
| 5 | Oracle | 0.003 | 0.073 | 0.073 | 0.005 | 0.284 | 0.952 |  | 0.001 | 0.044 | 0.044 | 0.002 | 0.172 | 0.948 |
|  | fJM-NCC | 0.002 | 0.073 | 0.074 | 0.005 | 0.288 | 0.952 |  | 0.001 | 0.045 | 0.046 | 0.002 | 0.176 | 0.940 |
|  | wJM-NCC | 0.003 | 0.074 | 0.074 | 0.006 | 0.290 | 0.948 |  | 0.001 | 0.047 | 0.047 | 0.002 | 0.182 | 0.952 |
|  | wJM-NCC(Fisher) | 0.003 | 0.072 | 0.074 | 0.006 | 0.284 | 0.945 |  | 0.001 | 0.044 | 0.047 | 0.002 | 0.172 | 0.930 |
|  | JM | -0.007 | 0.072 | 0.073 | 0.005 | 0.282 | 0.949 |  | -0.008 | 0.044 | 0.044 | 0.002 | 0.171 | 0.940 |
|  | CLR | -0.004 | 0.073 | 0.075 | 0.006 | 0.288 | 0.947 |  | -0.008 | 0.045 | 0.045 | 0.002 | 0.174 | 0.944 |

1. Control-to-case ratio, i.e., the number of controls per case in the NCC sub-cohort

2. Estimated standard error

3. Empirical standard error

4. Mean squared error

5. Average length of the 95% confidence intervals

6. Empirical coverage probability of the 95% confidence interval

**Table S3:** Performance of all methods for point and 95% confidence interval estimation of $\beta_{1}$ and $\beta_{2}$ under **Scenario 2** ($\beta_{1}=0.2$ and $\beta_{2}=0.1$) in Study 1.

|  |  | $\beta_{1}$ | | | | | |  | $\beta_{2}$ | | | | | |
| --- | --- | --- | --- | --- | --- | --- | --- | --- | --- | --- | --- | --- | --- | --- |
| **m^1^** | **Method** | **Bias** | **SE^2^** | **ESE^3^** | **MSE^4^** | **CI-L^5^** | **ECP^6^** |  | **Bias** | **SE** | **ESE** | **MSE** | **CI-L** | **ECP** |
| 1 | Oracle | -0.003 | 0.071 | 0.069 | 0.005 | 0.279 | 0.963 |  | 0.001 | 0.044 | 0.044 | 0.002 | 0.173 | 0.941 |
|  | fJM-NCC | 0.000 | 0.074 | 0.072 | 0.005 | 0.289 | 0.967 |  | 0.002 | 0.047 | 0.048 | 0.002 | 0.183 | 0.944 |
|  | wJM-NCC | -0.002 | 0.082 | 0.080 | 0.006 | 0.320 | 0.957 |  | 0.001 | 0.058 | 0.059 | 0.003 | 0.227 | 0.947 |
|  | wJM-NCC(Fisher) | -0.002 | 0.071 | 0.080 | 0.006 | 0.280 | 0.927 |  | 0.001 | 0.044 | 0.059 | 0.003 | 0.173 | 0.866 |
|  | JM | -0.044 | 0.071 | 0.070 | 0.007 | 0.277 | 0.903 |  | -0.038 | 0.044 | 0.044 | 0.003 | 0.172 | 0.866 |
|  | CLR | -0.019 | 0.098 | 0.098 | 0.010 | 0.385 | 0.953 |  | -0.006 | 0.059 | 0.059 | 0.004 | 0.230 | 0.945 |
| 3 | Oracle | 0.001 | 0.072 | 0.071 | 0.005 | 0.281 | 0.952 |  | 0.001 | 0.044 | 0.044 | 0.002 | 0.173 | 0.947 |
|  | fJM-NCC | 0.003 | 0.073 | 0.073 | 0.005 | 0.286 | 0.946 |  | 0.002 | 0.046 | 0.045 | 0.002 | 0.179 | 0.956 |
|  | wJM-NCC | 0.001 | 0.075 | 0.075 | 0.006 | 0.294 | 0.956 |  | 0.001 | 0.049 | 0.049 | 0.002 | 0.192 | 0.947 |
|  | wJM-NCC(Fisher) | 0.001 | 0.072 | 0.075 | 0.006 | 0.281 | 0.944 |  | 0.001 | 0.044 | 0.049 | 0.002 | 0.173 | 0.915 |
|  | JM | -0.019 | 0.071 | 0.071 | 0.005 | 0.278 | 0.952 |  | -0.018 | 0.044 | 0.044 | 0.002 | 0.172 | 0.928 |
|  | CLR | -0.016 | 0.078 | 0.077 | 0.006 | 0.305 | 0.952 |  | -0.007 | 0.047 | 0.048 | 0.002 | 0.185 | 0.946 |
| 5 | Oracle | 0.001 | 0.072 | 0.077 | 0.006 | 0.281 | 0.934 |  | 0.003 | 0.044 | 0.046 | 0.002 | 0.173 | 0.941 |
|  | fJM-NCC | 0.002 | 0.073 | 0.077 | 0.006 | 0.285 | 0.938 |  | 0.003 | 0.045 | 0.047 | 0.002 | 0.177 | 0.939 |
|  | wJM-NCC | 0.001 | 0.073 | 0.078 | 0.006 | 0.287 | 0.945 |  | 0.003 | 0.047 | 0.049 | 0.002 | 0.183 | 0.942 |
|  | wJM-NCC(Fisher) | 0.001 | 0.072 | 0.078 | 0.006 | 0.280 | 0.932 |  | 0.003 | 0.044 | 0.049 | 0.002 | 0.173 | 0.928 |
|  | JM | -0.013 | 0.071 | 0.075 | 0.006 | 0.278 | 0.930 |  | -0.009 | 0.044 | 0.045 | 0.002 | 0.171 | 0.934 |
|  | CLR | -0.017 | 0.073 | 0.079 | 0.006 | 0.287 | 0.923 |  | -0.005 | 0.045 | 0.046 | 0.002 | 0.176 | 0.942 |

1. Control-to-case ratio, i.e., the number of controls per case in the NCC sub-cohort

2. Estimated standard error

3. Empirical standard error

4. Mean squared error

5. Average length of the 95% confidence intervals

6. Empirical coverage probability of the 95% confidence interval

**Table S4**: Performance of all methods for point estimation of additional parameters under **Scenario 1**($\beta_{1}=\beta_{2}=0$.) in Study 1. Parameters include the fixed slope $\gamma$, standard deviation (log) of the random intercept $\theta$, standard deviation (log) of random error $\sigma$, and the fixed effect $\alpha$.

|  |  | $\gamma=0.1$ | | |  | $\log\left( \theta\right)=\log\surd2$ | | |  | $\log\left( \sigma\right)=0$ | | |  | $\alpha=-0.2$ | | |
| --- | --- | --- | --- | --- | --- | --- | --- | --- | --- | --- | --- | --- | --- | --- | --- | --- |
| **m^1^** | **Method** | **Bias** | **SE^2^** | **ESE^3^** |  | **Bias** | **SE** | **ESE** |  | **Bias** | **SE** | **ESE** |  | **Bias** | **SE** | **ESE** |
| 1 | Oracle | 0.000 | 0.033 | 0.033 |  | -0.005 | 0.008 | 0.009 |  | 0.001 | 0.004 | 0.004 |  | -0.004 | 0.101 | 0.100 |
|  | fJM-NCC | 0.003 | 0.111 | 0.109 |  | -0.008 | 0.027 | 0.029 |  | 0.001 | 0.013 | 0.013 |  | -0.004 | 0.101 | 0.100 |
|  | wJM-NCC | 0.005 | 0.144 | 0.138 |  | -0.007 | 0.038 | 0.039 |  | 0.000 | 0.017 | 0.017 |  | -0.004 | 0.141 | 0.103 |
|  | wJM-NCC(Fisher) | 0.005 | 0.033 | 0.138 |  | -0.007 | 0.008 | 0.039 |  | 0.000 | 0.004 | 0.017 |  | -0.004 | 0.101 | 0.103 |
|  | JM | 0.004 | 0.120 | 0.115 |  | 0.007 | / | 0.031 |  | 0.024 | / | 0.012 |  | 0.194 | 0.101 | 0.026 |
| 3 | Oracle | 0.000 | 0.033 | 0.032 |  | -0.005 | 0.008 | 0.009 |  | 0.001 | 0.004 | 0.004 |  | -0.001 | 0.101 | 0.103 |
|  | fJM-NCC | -0.002 | 0.078 | 0.082 |  | -0.005 | 0.020 | 0.020 |  | 0.001 | 0.009 | 0.009 |  | -0.001 | 0.101 | 0.103 |
|  | wJM-NCC | -0.004 | 0.086 | 0.086 |  | -0.005 | 0.022 | 0.022 |  | 0.001 | 0.010 | 0.010 |  | 0.000 | 0.115 | 0.105 |
|  | wJM-NCC(Fisher) | -0.004 | 0.033 | 0.086 |  | -0.005 | 0.008 | 0.022 |  | 0.001 | 0.004 | 0.010 |  | 0.000 | 0.101 | 0.105 |
|  | JM | -0.002 | 0.086 | 0.088 |  | 0.009 | / | 0.021 |  | 0.025 | / | 0.009 |  | 0.184 | 0.101 | 0.019 |
| 5 | Oracle | 0.000 | 0.033 | 0.033 |  | -0.005 | 0.008 | 0.009 |  | 0.001 | 0.004 | 0.004 |  | 0.000 | 0.101 | 0.100 |
|  | fJM-NCC | -0.001 | 0.064 | 0.067 |  | -0.006 | 0.016 | 0.017 |  | 0.001 | 0.008 | 0.008 |  | 0.000 | 0.101 | 0.100 |
|  | wJM-NCC | 0.001 | 0.068 | 0.070 |  | -0.006 | 0.018 | 0.018 |  | 0.001 | 0.008 | 0.008 |  | 0.000 | 0.109 | 0.101 |
|  | wJM-NCC(Fisher) | 0.001 | 0.033 | 0.070 |  | -0.006 | 0.008 | 0.018 |  | 0.001 | 0.004 | 0.008 |  | 0.000 | 0.101 | 0.101 |
|  | JM | -0.001 | 0.071 | 0.069 |  | 0.009 | / | 0.018 |  | 0.025 | / | 0.007 |  | 0.173 | 0.101 | 0.020 |

1. Control-to-case ratio, i.e., the number of controls per case in the NCC sub-cohort

2. Estimated standard error

3. Empirical standard error

**Table S5**: Performance of all methods for point estimation of additional parameters under **Scenario 2** ($\beta_{1}=0$ and $\beta_{2}=0.1$) in Study 1. Parameters include the fixed slope $\gamma$, standard deviation (log) of the random intercept $\theta$, standard deviation (log) of random error $\sigma$, and the fixed effect $\alpha$.

|  |  | $\gamma=0.1$ | | |  | $\log\left( \theta\right)=\log\surd2$ | | |  | $\log\left( \sigma\right)=0$ | | |  | $\alpha=-0.2$ | | |
| --- | --- | --- | --- | --- | --- | --- | --- | --- | --- | --- | --- | --- | --- | --- | --- | --- |
| **m^1^** | **Method** | **Bias** | **SE^2^** | **ESE^3^** |  | **Bias** | **SE** | **ESE** |  | **Bias** | **SE** | **ESE** |  | **Bias** | **SE** | **ESE** |
| 1 | Oracle | -0.001 | 0.033 | 0.034 |  | -0.005 | 0.008 | 0.009 |  | 0.001 | 0.004 | 0.004 |  | -0.002 | 0.101 | 0.098 |
|  | fJM-NCC | -0.002 | 0.111 | 0.113 |  | -0.007 | 0.027 | 0.028 |  | 0.000 | 0.013 | 0.013 |  | -0.002 | 0.101 | 0.098 |
|  | wJM-NCC | -0.002 | 0.144 | 0.136 |  | -0.008 | 0.038 | 0.039 |  | 0.001 | 0.017 | 0.018 |  | -0.002 | 0.141 | 0.100 |
|  | wJM-NCC(Fisher) | -0.002 | 0.033 | 0.136 |  | -0.008 | 0.008 | 0.039 |  | 0.001 | 0.004 | 0.018 |  | -0.002 | 0.101 | 0.100 |
|  | JM | 0.001 | 0.120 | 0.122 |  | 0.010 | / | 0.029 |  | 0.024 | / | 0.013 |  | 0.194 | 0.101 | 0.027 |
| 3 | Oracle | 0.001 | 0.033 | 0.032 |  | -0.005 | 0.008 | 0.009 |  | 0.001 | 0.004 | 0.004 |  | -0.002 | 0.101 | 0.101 |
|  | fJM-NCC | -0.002 | 0.078 | 0.077 |  | -0.007 | 0.020 | 0.020 |  | 0.001 | 0.009 | 0.009 |  | -0.002 | 0.101 | 0.101 |
|  | wJM-NCC | -0.005 | 0.086 | 0.084 |  | -0.007 | 0.022 | 0.022 |  | 0.001 | 0.010 | 0.010 |  | -0.002 | 0.115 | 0.102 |
|  | wJM-NCC(Fisher) | -0.005 | 0.033 | 0.084 |  | -0.007 | 0.008 | 0.022 |  | 0.001 | 0.004 | 0.010 |  | -0.002 | 0.101 | 0.102 |
|  | JM | -0.005 | 0.086 | 0.082 |  | 0.009 | / | 0.022 |  | 0.025 | / | 0.009 |  | 0.182 | 0.101 | 0.019 |
| 5 | Oracle | 0.001 | 0.033 | 0.033 |  | -0.005 | 0.008 | 0.009 |  | 0.001 | 0.004 | 0.004 |  | 0.002 | 0.101 | 0.097 |
|  | fJM-NCC | 0.000 | 0.064 | 0.063 |  | -0.006 | 0.016 | 0.017 |  | 0.001 | 0.008 | 0.008 |  | 0.002 | 0.101 | 0.097 |
|  | wJM-NCC | 0.000 | 0.068 | 0.067 |  | -0.005 | 0.018 | 0.017 |  | 0.001 | 0.008 | 0.008 |  | 0.002 | 0.109 | 0.098 |
|  | wJM-NCC(Fisher) | 0.000 | 0.033 | 0.067 |  | -0.005 | 0.008 | 0.017 |  | 0.001 | 0.004 | 0.008 |  | 0.002 | 0.101 | 0.098 |
|  | JM | 0.000 | 0.071 | 0.068 |  | 0.010 | / | 0.018 |  | 0.025 | / | 0.007 |  | 0.173 | 0.101 | 0.021 |

1. Control-to-case ratio, i.e., the number of controls per case in the NCC sub-cohort

2. Estimated standard error

3. Empirical standard error

**Table S6**: Performance of all methods for point estimation of additional parameters under **Scenario 2**($\beta_{1}=0.1$ and $\beta_{2}=0.1$) in Study 1. Parameters include the fixed slope $\gamma$, standard deviation (log) of the random intercept $\theta$, standard deviation (log) of random error $\sigma$, and the fixed effect $\alpha$.

|  |  | $\gamma=0.1$ | | |  | $\log\left( \theta\right)=\log\surd2$ | | |  | $\log\left( \sigma\right)=0$ | | |  | $\alpha=-0.2$ | | |
| --- | --- | --- | --- | --- | --- | --- | --- | --- | --- | --- | --- | --- | --- | --- | --- | --- |
| **m^1^** | **Method** | **Bias** | **SE^2^** | **ESE^3^** |  | **Bias** | **SE** | **ESE** |  | **Bias** | **SE** | **ESE** |  | **Bias** | **SE** | **ESE** |
| 1 | Oracle | 0.001 | 0.033 | 0.034 |  | -0.004 | 0.008 | 0.009 |  | 0.001 | 0.004 | 0.004 |  | 0.004 | 0.101 | 0.102 |
|  | fJM-NCC | 0.001 | 0.111 | 0.112 |  | -0.006 | 0.027 | 0.029 |  | 0.000 | 0.013 | 0.013 |  | 0.004 | 0.101 | 0.102 |
|  | wJM-NCC | 0.001 | 0.144 | 0.141 |  | -0.005 | 0.038 | 0.038 |  | 0.001 | 0.017 | 0.018 |  | 0.005 | 0.142 | 0.105 |
|  | wJM-NCC(Fisher) | 0.001 | 0.033 | 0.141 |  | -0.005 | 0.008 | 0.038 |  | 0.001 | 0.004 | 0.018 |  | 0.005 | 0.101 | 0.105 |
|  | JM | 0.004 | 0.120 | 0.120 |  | 0.011 | / | 0.033 |  | 0.024 | / | 0.013 |  | 0.193 | 0.101 | 0.042 |
| 3 | Oracle | -0.001 | 0.033 | 0.032 |  | -0.005 | 0.008 | 0.009 |  | 0.001 | 0.004 | 0.004 |  | 0.001 | 0.101 | 0.101 |
|  | fJM-NCC | -0.001 | 0.078 | 0.080 |  | -0.005 | 0.020 | 0.020 |  | 0.002 | 0.009 | 0.010 |  | 0.001 | 0.101 | 0.101 |
|  | wJM-NCC | -0.004 | 0.086 | 0.088 |  | -0.005 | 0.022 | 0.022 |  | 0.002 | 0.010 | 0.011 |  | 0.001 | 0.115 | 0.102 |
|  | wJM-NCC(Fisher) | -0.004 | 0.033 | 0.088 |  | -0.005 | 0.008 | 0.022 |  | 0.002 | 0.004 | 0.011 |  | 0.001 | 0.101 | 0.102 |
|  | JM | -0.003 | 0.086 | 0.086 |  | 0.011 | / | 0.022 |  | 0.026 | / | 0.009 |  | 0.182 | 0.101 | 0.020 |
| 5 | Oracle | 0.000 | 0.033 | 0.032 |  | -0.005 | 0.008 | 0.009 |  | 0.001 | 0.004 | 0.004 |  | 0.000 | 0.101 | 0.104 |
|  | fJM-NCC | 0.004 | 0.064 | 0.067 |  | -0.005 | 0.016 | 0.017 |  | 0.001 | 0.008 | 0.008 |  | 0.000 | 0.101 | 0.104 |
|  | wJM-NCC | 0.003 | 0.068 | 0.070 |  | -0.004 | 0.018 | 0.018 |  | 0.001 | 0.008 | 0.008 |  | 0.000 | 0.109 | 0.106 |
|  | wJM-NCC(Fisher) | 0.003 | 0.033 | 0.070 |  | -0.004 | 0.008 | 0.018 |  | 0.001 | 0.004 | 0.008 |  | 0.000 | 0.101 | 0.106 |
|  | JM | 0.004 | 0.071 | 0.071 |  | 0.011 | / | 0.018 |  | 0.026 | / | 0.007 |  | 0.173 | 0.101 | 0.022 |

1. Control-to-case ratio, i.e., the number of controls per case in the NCC sub-cohort

2. Estimated standard error

3. Empirical standard error

**Table S7**: Performance of all methods for point estimation of additional parameters under **Scenario 2**($\beta_{1}=0.2$ and $\beta_{2}=0.1$) in Study 1. Parameters include the fixed slope $\gamma$, standard deviation (log) of the random intercept $\theta$, standard deviation (log) of random error $\sigma$, and the fixed effect $\alpha$.

|  |  | $\gamma=0.1$ | | |  | $\log\left( \theta\right)=\log\surd2$ | | |  | $\log\left( \sigma\right)=0$ | | |  | $\alpha=-0.2$ | | |
| --- | --- | --- | --- | --- | --- | --- | --- | --- | --- | --- | --- | --- | --- | --- | --- | --- |
| **m^1^** | **Method** | **Bias** | **SE^2^** | **ESE^3^** |  | **Bias** | **SE** | **ESE** |  | **Bias** | **SE** | **ESE** |  | **Bias** | **SE** | **ESE** |
| 1 | Oracle | 0.001 | 0.033 | 0.032 |  | -0.005 | 0.008 | 0.009 |  | 0.001 | 0.004 | 0.004 |  | -0.004 | 0.101 | 0.098 |
|  | fJM-NCC | -0.001 | 0.110 | 0.112 |  | -0.006 | 0.027 | 0.028 |  | 0.001 | 0.013 | 0.013 |  | -0.005 | 0.101 | 0.098 |
|  | wJM-NCC | -0.003 | 0.144 | 0.145 |  | -0.004 | 0.038 | 0.039 |  | 0.000 | 0.017 | 0.018 |  | -0.006 | 0.142 | 0.102 |
|  | wJM-NCC(Fisher) | -0.003 | 0.033 | 0.145 |  | -0.004 | 0.008 | 0.039 |  | 0.000 | 0.004 | 0.018 |  | -0.006 | 0.101 | 0.102 |
|  | JM | -0.001 | 0.121 | 0.119 |  | 0.014 | / | 0.031 |  | 0.025 | / | 0.012 |  | 0.190 | 0.101 | 0.028 |
| 3 | Oracle | -0.001 | 0.033 | 0.033 |  | -0.005 | 0.008 | 0.009 |  | 0.001 | 0.004 | 0.004 |  | 0.001 | 0.101 | 0.100 |
|  | fJM-NCC | 0.003 | 0.077 | 0.078 |  | -0.006 | 0.020 | 0.020 |  | 0.001 | 0.009 | 0.009 |  | 0.001 | 0.101 | 0.100 |
|  | wJM-NCC | 0.001 | 0.085 | 0.086 |  | -0.006 | 0.022 | 0.023 |  | 0.001 | 0.010 | 0.010 |  | 0.002 | 0.115 | 0.102 |
|  | wJM-NCC(Fisher) | 0.001 | 0.033 | 0.086 |  | -0.006 | 0.008 | 0.023 |  | 0.001 | 0.004 | 0.010 |  | 0.002 | 0.101 | 0.102 |
|  | JM | 0.002 | 0.086 | 0.084 |  | 0.012 | */* | 0.021 |  | 0.025 | / | 0.009 |  | 0.183 | 0.101 | 0.021 |
| 5 | Oracle | -0.001 | 0.033 | 0.034 |  | -0.005 | 0.008 | 0.009 |  | 0.001 | 0.004 | 0.004 |  | 0.000 | 0.101 | 0.100 |
|  | fJM-NCC | 0.000 | 0.064 | 0.065 |  | -0.005 | 0.016 | 0.016 |  | 0.001 | 0.008 | 0.007 |  | 0.000 | 0.101 | 0.100 |
|  | wJM-NCC | 0.000 | 0.068 | 0.068 |  | -0.004 | 0.018 | 0.017 |  | 0.001 | 0.008 | 0.008 |  | 0.000 | 0.109 | 0.102 |
|  | wJM-NCC(Fisher) | 0.000 | 0.033 | 0.068 |  | -0.004 | 0.008 | 0.017 |  | 0.001 | 0.004 | 0.008 |  | 0.000 | 0.101 | 0.102 |
|  | JM | 0.000 | 0.071 | 0.071 |  | 0.012 | / | 0.018 |  | 0.026 | / | 0.007 |  | 0.173 | 0.101 | 0.022 |

1. Control-to-case ratio, i.e., the number of controls per case in the NCC sub-cohort

2. Estimated standard error

3. Empirical standard error

**Table S8**: Performance of all methods for point estimation of additional parameters under **Scenario 2**($\beta_{1}=0.3$ and $\beta_{2}=0.1$) in Study 1. Parameters include the fixed slope $\gamma$, standard deviation (log) of the random intercept $\theta$, standard deviation (log) of random error $\sigma$, and the fixed effect $\alpha$.

|  |  | $\gamma=0.1$ | | |  | $\log\left( \theta\right)=\log\surd2$ | | |  | $\log\left( \sigma\right)=0$ | | |  | $\alpha=-0.2$ | | |
| --- | --- | --- | --- | --- | --- | --- | --- | --- | --- | --- | --- | --- | --- | --- | --- | --- |
| **m^1^** | **Method** | **Bias** | **SE^2^** | **ESE^3^** |  | **Bias** | **SE** | **ESE** |  | **Bias** | **SE** | **ESE** |  | **Bias** | **SE** | **ESE** |
| 1 | Oracle | 0.000 | 0.033 | 0.033 |  | -0.004 | 0.008 | 0.008 |  | 0.001 | 0.004 | 0.004 |  | -0.004 | 0.101 | 0.097 |
|  | fJM-NCC | 0.007 | 0.108 | 0.104 |  | -0.006 | 0.027 | 0.027 |  | 0.000 | 0.013 | 0.012 |  | -0.004 | 0.101 | 0.097 |
|  | wJM-NCC | 0.004 | 0.144 | 0.137 |  | -0.005 | 0.038 | 0.038 |  | 0.000 | 0.017 | 0.016 |  | -0.003 | 0.143 | 0.101 |
|  | wJM-NCC(Fisher) | 0.004 | 0.033 | 0.137 |  | -0.005 | 0.008 | 0.038 |  | 0.000 | 0.004 | 0.016 |  | -0.003 | 0.101 | 0.101 |
|  | JM | 0.008 | 0.121 | 0.113 |  | 0.019 | / | 0.029 |  | 0.025 | / | 0.012 |  | 0.191 | 0.101 | 0.089 |
| 3 | Oracle | 0.001 | 0.033 | 0.031 |  | -0.004 | 0.008 | 0.009 |  | 0.001 | 0.004 | 0.004 |  | 0.000 | 0.101 | 0.100 |
|  | fJM-NCC | 0.001 | 0.077 | 0.076 |  | -0.005 | 0.020 | 0.020 |  | 0.001 | 0.009 | 0.009 |  | 0.000 | 0.101 | 0.100 |
|  | wJM-NCC | 0.002 | 0.085 | 0.084 |  | -0.005 | 0.022 | 0.022 |  | 0.001 | 0.010 | 0.010 |  | 0.000 | 0.116 | 0.102 |
|  | wJM-NCC(Fisher) | 0.002 | 0.033 | 0.084 |  | -0.005 | 0.008 | 0.022 |  | 0.001 | 0.004 | 0.010 |  | 0.000 | 0.101 | 0.102 |
|  | JM | 0.000 | 0.086 | 0.083 |  | 0.016 | / | 0.021 |  | 0.026 | / | 0.009 |  | 0.182 | 0.101 | 0.023 |
| 5 | Oracle | 0.000 | 0.033 | 0.033 |  | -0.005 | 0.008 | 0.009 |  | 0.001 | 0.004 | 0.004 |  | 0.001 | 0.101 | 0.102 |
|  | fJM-NCC | 0.002 | 0.064 | 0.064 |  | -0.005 | 0.016 | 0.016 |  | 0.000 | 0.008 | 0.008 |  | 0.001 | 0.101 | 0.102 |
|  | wJM-NCC | 0.000 | 0.068 | 0.068 |  | -0.005 | 0.018 | 0.017 |  | 0.000 | 0.008 | 0.008 |  | 0.002 | 0.109 | 0.106 |
|  | wJM-NCC(Fisher) | 0.000 | 0.033 | 0.068 |  | -0.005 | 0.008 | 0.017 |  | 0.000 | 0.004 | 0.008 |  | 0.002 | 0.101 | 0.106 |
|  | JM | 0.002 | 0.071 | 0.070 |  | 0.014 | / | 0.018 |  | 0.025 | / | 0.007 |  | 0.174 | 0.101 | 0.024 |

1. Control-to-case ratio, i.e., the number of controls per case in the NCC sub-cohort

2. Estimated standard error

3. Empirical standard error

**Table S9:** Estimation results and hypothesis testing p-values for the species that associated with appearance of IAA-first ($\beta_{1}$) and GADA-first ($\beta_{2}$) identified by various methods.

| **Species** | **fJM-NCC** | **wJM-NCC** | **JM** | **CLR** |
| --- | --- | --- | --- | --- |
| *Megasphaera elsdenii* | (-40.50, -48.40)^1^  (< 0.001, < 0.001)^2^ | (-38.90, -46.40)  (1, 1) | (-4.921, -9.273)  (1, 1) | (8.97, 15.64)  (1, 1) |
| *Bifidobacterium dentium* | (4.75, 4.68)  (< 0.001, < 0.001) | (-5.98, -5.05)  (0.199, 1.00) | (-6.148, -1.03)  (1, 1) | (-10.66, -2.26)  (0.639, 1) |
| *Lactobacillus delbrueckii* | (-80.20, 22.70)  (0.001, 1) | (-58.80, 42.20)  (0.322, 1) | (-28.202, 15.437)  (1, 1) | (67.25, -11.26)  (1, 1) |
| *Bacteroides heparinolyticus* | (-35.80, 5.20)  (0.001, 1) | (-35.70, 5.25)  (0.040, 1) | (-6.956, -2.791)  (1, 1) | (11.06, 14.79)  (1, 1) |
| *Ethanoligenens harbinense* | (-43.3, 26.7)  (0.002, 1) | (-30.9, 30.7)  (0.138, 1) | (-10.896, 11.979)  (1, 1) | (105.518, 87.925)  (0.153, 1) |
| *Acidaminococcus intestini* | (-36.7, -16)  (0.002, 1) | (-36.1, -15.2)  (1, 1) | (0.254, 12.157)  (1, 1) | (22.051, -5.407)  (1, 1) |
| *Bacteroides sp. A1C1* | (-7, 0.412)  (0.003, 1) | (-5.59, 1.1)  (0.082, 1) | (-1.957, -0.816)  (1, 1) | (-1.161, -3.188)  (1, 1) |
| *Escherichia marmotae* | (116, -52.6)  (0.004, 1) | (116, -52.8)  (0.118, 1) | (13.602, -10.881)  (1, 1) | (23.832, -8.44)  (1, 1) |
| *Oscillibacter valericigenes* | (-31.9, 24.4)  (0.012, 1) | (-26.4, 24.6)  (0.060, 1) | (-7.73, 9.426)  (1, 1) | (78.761, 46.646)  (0.401, 1) |
| *Corynebacterium argentoratense* | (-50.8, -2.79)  (0.013, 1) | (-50.5, -2.53)  (0.225, 1) | (-13.936, -11.795)  (1, 1) | (10.472, -15.795)  (1, 1) |
| *Gordonibacter urolithinfaciens* | (-34.8, 10.7)  (0.013, 1) | (-34.7, 10.6)  (0.050, 1) | (-17.646, 5.16)  (1, 1) | (4.748, -4.489)  (1, 1) |
| *Ruminococcus albus* | (-31.6, 23.6)  (0.014, 1) | (-23.2, 27.2)  (0.543, 1) | (-3.988, 13.923)  (1, 1) | (98.702, 69.7)  (0.028, 1) |
| *Ruminococcaceae bacterium CPB6* | (-34, 27.8)  (0.014, 1) | (-26.2, 29.6)  (0.242, 1) | (-6.997, 14.125)  (1, 1) | (107.033, 79.485)  (0.024, 1) |
| *Caproiciproducens sp. NJN-50* | (-35, 30.4)  (0.014, 1) | (-28.9, 31.3)  (0.091, 1) | (-9.553, 16.332)  (1, 1) | (91.71, 89.276)  (0.138, 1) |
| *Paraprevotella xylaniphila* | (-29.7, 17.6)  (0.015, 1) | (-28.7, 17.9)  (0.116, 1) | (-2.096, 7.808)  (1, 1) | (14.925, 10.281)  (1, 1) |
| *Bacillus cereus* | (-101, -86.2)  (0.019, 1) | (-100, -86.1)  (0.048, 1) | (-2.504, -0.174)  (1, 1) | (-1.47, 136.413)  (1, 1) |
| *Bacteroides intestinalis* | (-18.4, 0.944)  (0.024, 1) | (-10.9, 6)  (1, 1) | (4.56, 1.705)  (1, 1) | (10.539, 7.884)  (1, 1) |
| *Escherichia albertii* | (77.2, -41.4)  (0.026, 1) | (56.4, -54.7)  (1, 1) | (25.474, -83.371)  (1, 1) | (-0.466, -78.615)  (1, 1) |
| *Alistipes finegoldii* | (-7.84, 4.15)  (0.029, 1) | (-7.29, 4.35)  (0.061, 1) | (-2.197, 0.811)  (1, 1) | (2.133, 0.458)  (1, 1) |
| *Ruminococcus bicirculans* | (-6.59, 6.16)  (0.033, 1) | (-5.63, 7.24)  (0.281, 1) | (0.4, 6.709)  (1, 1) | (14.039, 4.357)  (0.729, 1) |
| *Christensenella sp. Marseille-P3954* | (-32.3, 23.9)  (0.033, 1) | (-32, 23.9)  (0.012, 1) | (-6.721, 6.401)  (1, 1) | (88.909, 62.8)  (0.168, 1) |
| *Streptococcus thermophilus* | (-16.8, 2.91)  (0.033, 1) | (-16.5, 2.77)  (0.088, 1) | (-6.561, 4.14)  (1, 1) | (0.081, -3.191)  (1, 1) |
| *Corynebacterium variabile* | (-314, -362)  (0.033, 0.315) | (-314, -362)  (0.003, 0.375) | (-65.257, -46.172)  (1, 1) | (57.346, -97.577)  (1, 1) |
| *Ruminococcus champanellensis* | (-23.3, 22.1)  (0.038, 1) | (-23.1, 22.2)  (0.027, 1) | (-4, 009, 11.629)  (1, 1) | (77.238, 34.679)  (0.040, 1) |
| *Alistipes sp. 5CBH24* | (-29, 26.6)  (0.043, 1) | (-29, 26.4)  (0.008, 1) | (-10.96, 15.563)  (1, 1) | (19.585, 7.32)  (1, 1) |
| *Flintibacter sp. KGMB00164* | (-18.7, 13.8)  (0.045, 1) | (-15.9, 13.9)  (0.156, 1) | (-3.158, 2.082)  (1, 1) | (50.723, 18.737)  (0.282, 1) |
| *Bifidobacterium* | (-1.3, 0.059)  (0.059, 1) | (0.491, 1.76)  (1, 1) | (0.461, -0.988)  (1, 1) | (2.064, -0.768)  (1, 1) |
| *Clostridiales bacterium CCNA10* | (-10.2, 7.09)  (0.067, 1) | (-8.69, 7.02)  (0.132, 1) | (0.181, 2.689)  (1, 1) | (31.273, 17.425)  (0.087, 1) |
| *Ruminococcus sp. JE7A12* | (-11.1, 9.24)  (0.073, 1) | (-8.77, 11.7)  (1, 1) | (0.983, 7.228)  (1, 1) | (25.473, 20.154)  (0.591, 1) |
| *Veillonella parvula* | (6.63, -5.88)  (0.075, 1) | (6.08, -6.16)  (0.354, 1) | (1.789, -5.852)  (1, 1) | (-3.582, -4.292)  (1, 1) |
| *Bifidobacterium breve* | (1.35, 0.454)  (0.075, 1) | (0.968, 0.131)  (1, 1) | (-0.092, 0.821)  (1, 1) | (-0.84, 0.552)  (1, 1) |
| *Bacteroides dorei* | (-3.41, -1.62)  (0.075, 1) | (-2.04, -0.081)  (1, 1) | (-0.059, -0.479)  (1, 1) | (-0.878, 1.489)  (1, 1) |
| *Intestinimonas butyriciproducens* | (-25.9, 21)  (0.084, 1) | (-25.6, 21.2)  (0.083, 1) | (-2.087, 11.899)  (1, 1) | (93.448, 37.092)  (0.039, 1) |
| *Adlercreutzia equolifaciens* | (-18.5, 11.8)  (0.090, 1) | (-18.4, 11.8)  (0.208, 1) | (-3.929, -0.201)  (1, 1) | (31.881, 10.543)  (1, 1) |
| *Paeniclostridium sordellii* | (-43.9, 19.9)  (0.093, 1) | (-21.9, 28.6)  (1, 1) | (-0.682, 17.569)  (1, 1) | (40.45, 70.657)  (1, 1) |
| *Roseburia hominis* | (-7.71, 6)  (0.095, 1) | (-5.89, 6.82)  (1, 1) | (1.484, 4.686)  (1, 1) | (16.917, 18.309)  (0.596, 1) |
| *Collinsella aerofaciens* | (2.85, 7.19)  (0.276, <0.001) | (-0.603, 3.86)  (1, 0.789) | (-0.33, 2.973)  (1, 1) | (0.395, 5.9)  (1, 1) |
| *Lactobacillus paracasei* | (-11.4, -22.3)  (0.242, 0.002) | (3.26, -24.7)  (1, 1) | (-2.04, -20.649)  (1, 1) | (-7.275, -19.401)  (1, 1) |
| *Alistipes sp. 5CPEGH6* | (-23.8, 20)  (0.170, 1) | (-23.6, 19.9)  (0.098, 1) | (-6.369, 6.274)  (1, 1) | (50.942, 3.761)  (1, 1) |
| *[Eubacterium] sulci* | (-29.8, 12.2)  (1, 1) | (-39.7, 61.1)  (1, 1) | (-4.844, 35.316)  (1, 1) | (167.44, 122.065)  (0.009, 1) |
| *Lachnospiraceae bacterium GAM79* | (-6.4, 5.56)  (0.134, 1) | (-4.76, 6.91)  (1, 1) | (2.332, 5.229)  (1, 1) | (19.413, 10.612)  (0.020, 1) |
| *Turicibacter sp. H121* | (-15.9, 14.2)  (1, 1) | (-10.4, 17.2)  (1, 1) | (5.782, 12.909)  (1, 1) | (48.578, 32.014)  (0.048, 1) |
| *Anaerobutyricum hallii* | (-5.14, 4.31)  (0.566, 1) | (-3.79, 5.02)  (1, 1) | (2.13, 2.053)  (1, 1) | (17.125, 13.296)  (0.062, 1) |
| *Campylobacter jejuni* | (-13.4, 0.894)  (1, 1) | (-13.4, 1.1)  (1, 1) | (16.326, -15.286)  (1, 1) | (90.038, -28.752)  (0.062, 1) |

^1^ The point estimates and ^2^ the hypothesis testing p-values of ($\beta_{1}$, $\beta_{2}$) for the null hypothesis $H_{0}:\beta_{1}=0$ and $H_{0}:\beta_{2}=0$ respectively. p-value adjustments were conducted for $\beta_{1}$ and $\beta_{2}$ separately.

## **S8. Supplementary figures**


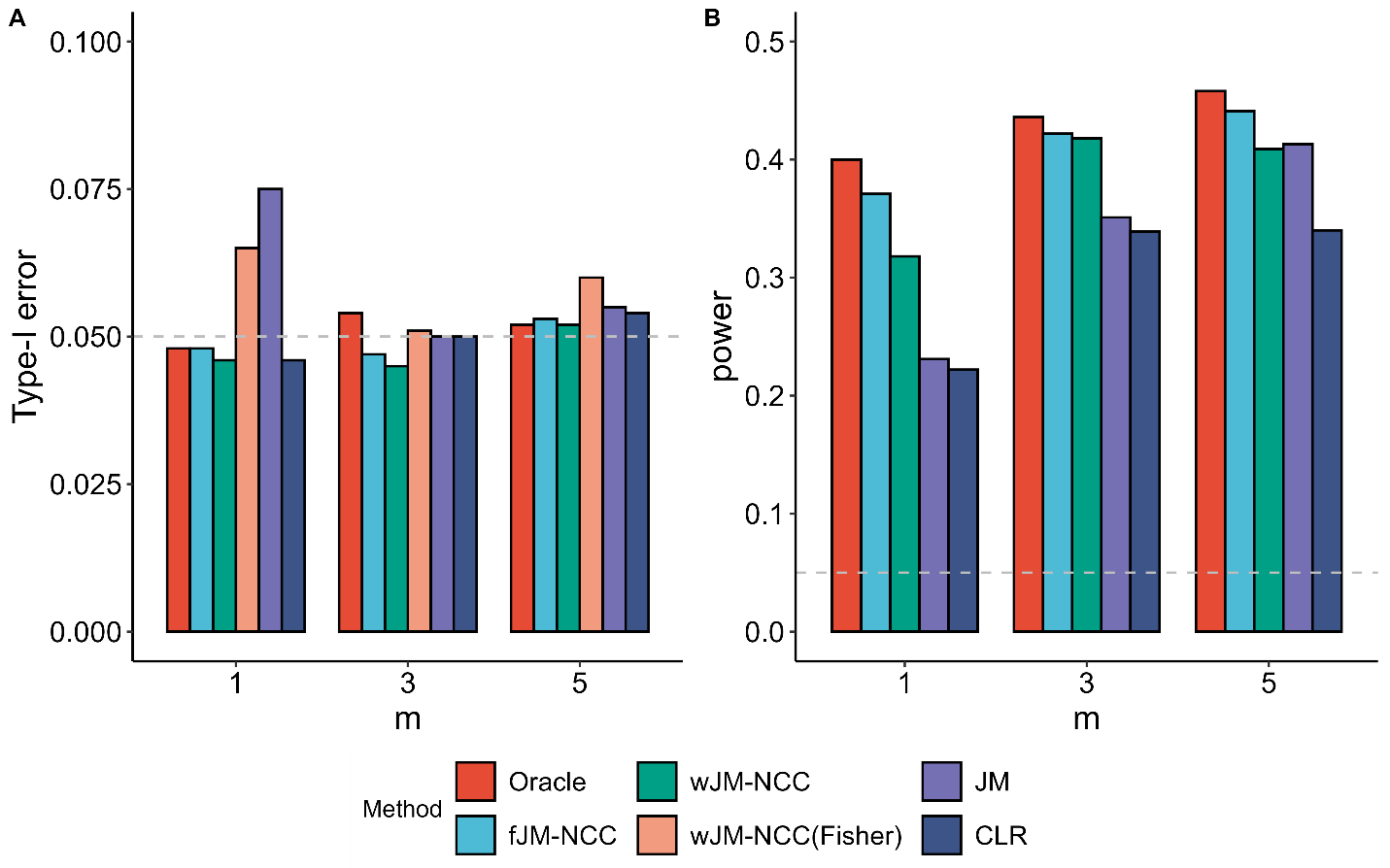


**Fig. S1**: Statistical testing performance of all considered methods for testing $H_{0}:\beta_{1}=0$ under $\beta_{1}=\beta_{2}=0$ and $\beta_{1}=\beta_{2}=0.1$, $m=1, 3, 5$, Study 2. **(A)**: Empirical Type-I error rates of all methods. **(B):** Statistical power of all methods, excluding wJM-NCC(Fisher) due to inflated Type-I error.


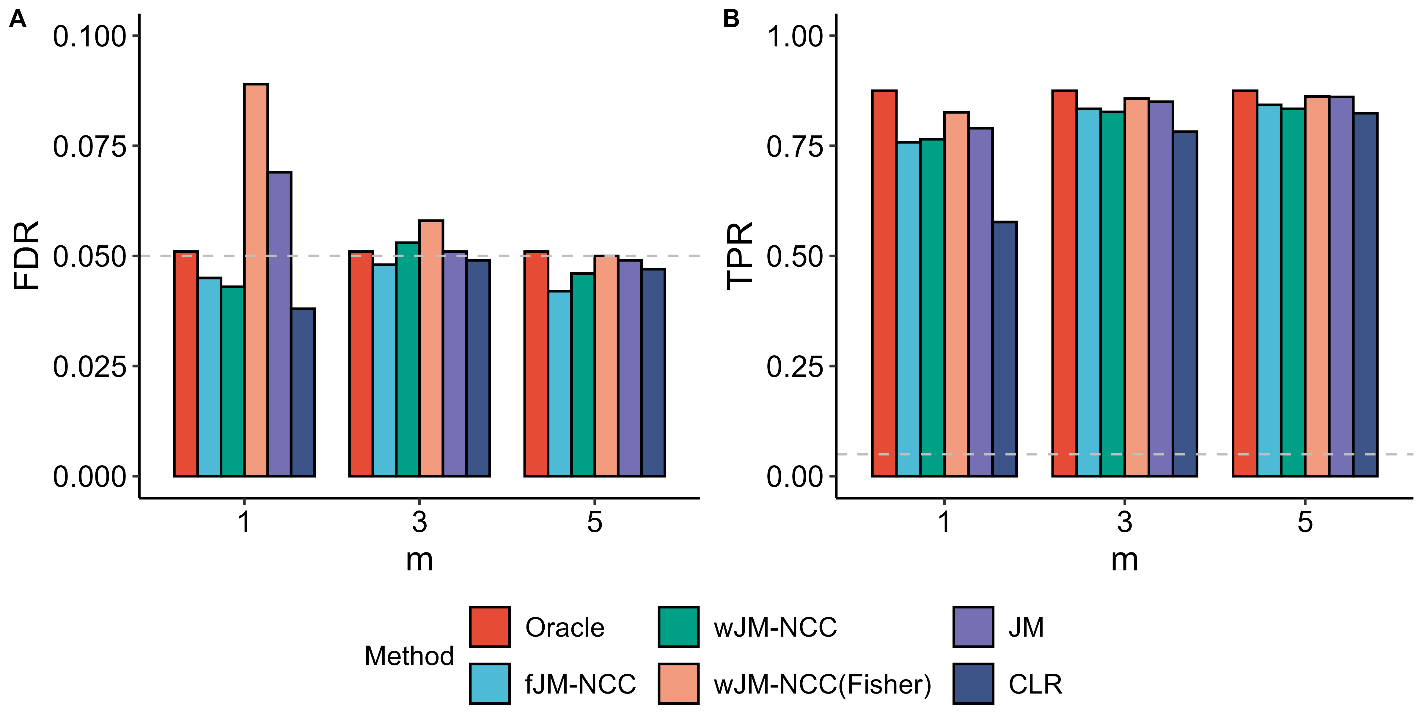


**Fig. S2:** Comparison of overall FDR (**A**) and TPR (**B**) across different methods for testing $(\beta_{1}, \beta_{2})$ , at FDR threshold of 0.05, $m=1, 3,$ and 5, Study 3.


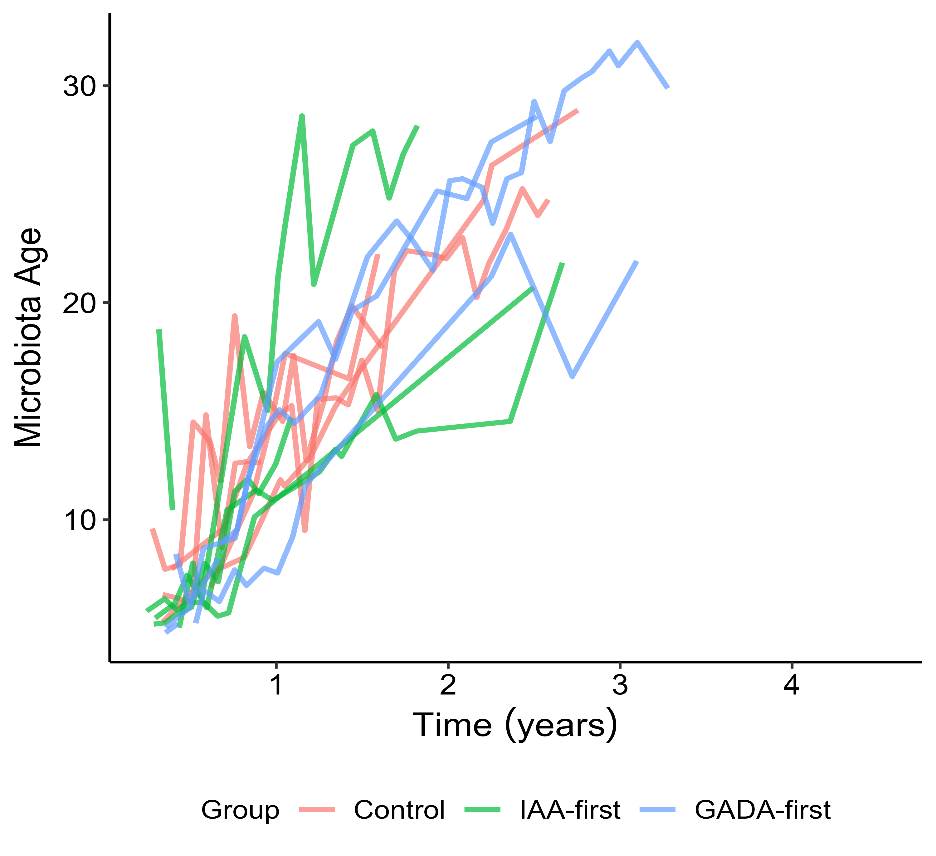


**Fig. S3:** Longitudinal trajectories for Microbiota age in the Control, IAA-first, and GADA-first group*.* Each line represents an individual participant.


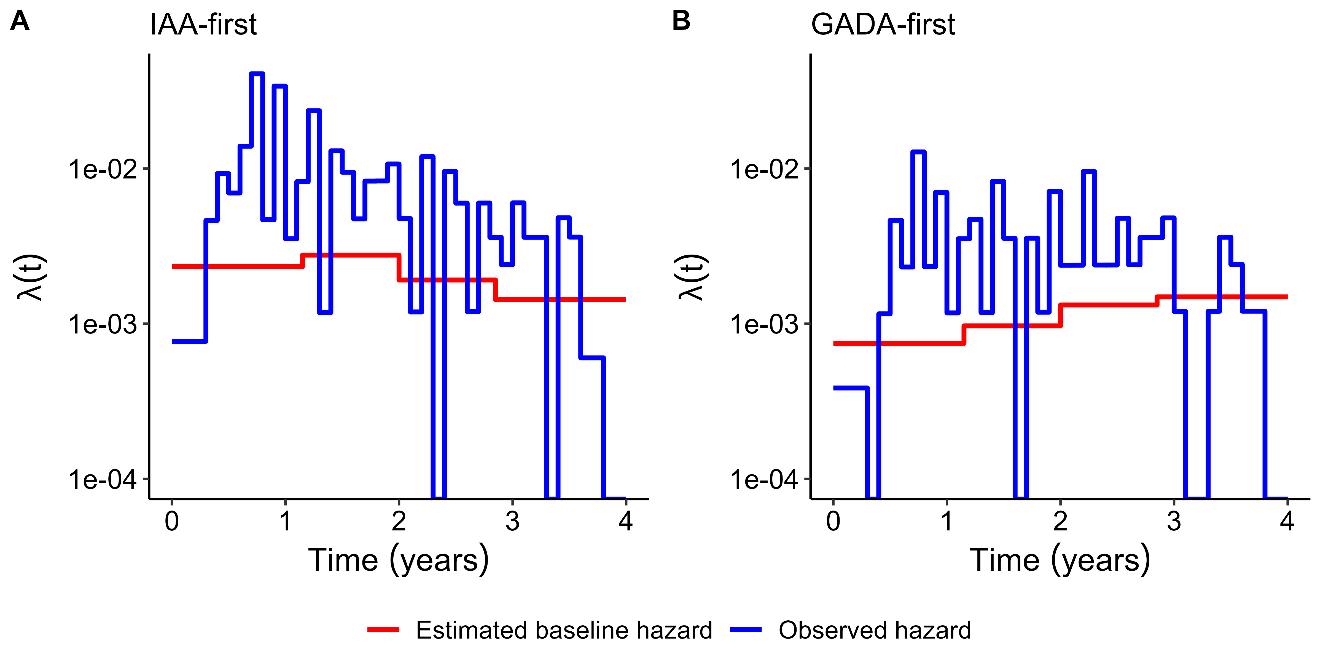


**Fig. S4:** Piecewise-constant baseline hazards estimated by fJM-NCC (red) and observed hazards from Nelson-Aalen estimator (blue) for Microbiota Age. **(A)** IAA-first event; **(B)** GADA-first event.

## **References**

Jiang J, Nguyen T. Linear and generalized linear mixed models and their applications (Vol. 1). *New York: Springer*, 2007.

Williamson PR, Kolamunnage‐Dona R, Philipson P, Marson AG. Joint modelling of longitudinal and competing risks data. *Statistics in medicine*, 2008; 27: 6426-6438.

Rizopoulos D. JM: An R package for the joint modelling of longitudinal and time-to-event data. *Journal of statistical software*, 2010; 35: 1-33.

Norris JM et al. Plasma 25-hydroxyvitamin D concentration and risk of islet autoimmunity. *Diabetes*, 2018; 67: 146-154.

Lee HS, Lynch KF, Krischer JP, TEDDY Study Group. Nested case‐control data analysis using weighted conditional logistic regression in The Environmental Determinants of Diabetes in the Young (TEDDY) study: A novel approach. *Diabetes/metabolism research and reviews*, 2020; 36: e3204.

Auchtung TA et al. Temporal changes in gastrointestinal fungi and the risk of autoimmunity during early childhood: the TEDDY study. *Nature Communications*, 2022; 13: 3151.

Gail MH, Lubin JH, Rubinstein LV. Likelihood calculations for matched case-control studies and survival studies with tied death times*. Biometrika*, 1981; 703-707.

Lee HS *et al*. Biomarker discovery study design for type 1 diabetes in The Environmental Determinants of Diabetes in the Young (TEDDY) study. *Diabetes/metabolism research and reviews*, 2014); 30: 424-434.

Rundle A, Ahsan H, Vineis P. Better cancer biomarker discovery through better study design. *European journal of clinical investigation*, 2012; 42: 1350-1359.

Rundle AG, Vineis P, Ahsan H. Design options for molecular epidemiology research within cohort studies. *Cancer Epidemiology Biomarkers & Prevention*, 2005; 14: 1899-1907.

Stewart C J *et al*. Temporal development of the gut microbiome in early childhood from the TEDDY study. *Nature*, 2018; 562: 583-588.

Subramanian S *et al*. Persistent gut microbiota immaturity in malnourished Bangladeshi children. *Nature*, 2014; 510: 417-421.

Ma, Siyuan, *et al*. A statistical model for describing and simulating microbial community profiles. *PLoS computational biology* 17.9 (2021): e1008913.
